# The Advance of CRISPR-Cas9-Based and NIR/CRISPR-Cas9-Based Imaging System

**DOI:** 10.3389/fchem.2021.786354

**Published:** 2021-12-16

**Authors:** Huanhuan Qiao, Jieting Wu, Xiaodong Zhang, Jian Luo, Hao Wang, Dong Ming

**Affiliations:** ^1^ Functional Materials Laboratory, Institute of Medical Engineering and Translational Medicine, Tianjin University, Tianjin, China; ^2^ Palo Alto Veterans Institute for Research, VA Palo Alto Health Care System, Palo Alto, CA, United States; ^3^ Department of Neurology and Neurological Sciences, School of Medicine, Stanford University, Stanford, CA, United States

**Keywords:** CRISPR-Cas9, NIR imaging, NIR fluorophores, CRISPR-Cas9-based imaging system, NIR/CRISPR-Cas9-based imaging system

## Abstract

The study of different genes, chromosomes and the spatiotemporal relationship between them is of great significance in the field of biomedicine. CRISPR-Cas9 has become the most widely used gene editing tool due to its excellent targeting ability. In recent years, a series of advanced imaging technologies based on Cas9 have been reported, providing fast and convenient tools for studying the sites location of genome, RNA, and chromatin. At the same time, a variety of CRISPR-Cas9-based imaging systems have been developed, which are widely used in real-time multi-site imaging *in vivo*. In this review, we summarized the component and mechanism of CRISPR-Cas9 system, overviewed the NIR imaging and the application of NIR fluorophores in the delivery of CRISPR-Cas9, and highlighted advances of the CRISPR-Cas9-based imaging system. In addition, we also discussed the challenges and potential solutions of CRISPR-Cas9-based imaging methods, and looked forward to the development trend of the field.

## 1 Introduction

From Zinc Finger Nucleases (ZFN) to transcription activator-like Effector Nucleases (TALEN), and then to Clustered Regularly Interspaced Short Palindromic Repeats (CRISPR) system, gene editing technology has been continuously explored and developed (Rui et al., 2019; Li H. et al., 2020; Kwon et al., 2021). Among them, the CRISPR-Cas9 system has become a revolutionary tool in the field of gene editing with its advantages of high efficiency, simple operation, and wide species adaptability (Pickar-Oliver and Gersbach, 2019; Yang et al., 2019). This emerging genome editing system can achieve precise knockout and insertion of specific gene sequences and even the genome of the entire organism, which is also known as “gene scissors” (Jiang and Doudna, 2017).

The development of genome editing allowed the manipulation of any gene in a variety of species and tissues (Hille et al., 2018). However, there was still a lack of accurate understanding of the relationship between operating gene and protein or phenotype. With the increasing interest in noninvasive imaging *in vivo*, the combination of CRISPR-Cas9 system and optical imaging technology has been studied to a certain extent. A robust CRISPR-Cas9-based imaging system could contribute to us new insights into how the structure and dynamics of molecules influence cell function under normal and abnormal status, which was not easily attainable by current biochemistry-based tools. Therefore, more efforts have been devoted to developing strategies that enable direct visualization of individual molecules by using of CRISPR-Cas9-based imaging system (Fu et al., 2016; Shao et al., 2016; Guo et al., 2019).

In this up-to-date review, we summarized a variety of CRISPR-Cas9 systems that have been developed for imaging of gene loci in living cells, especially in combination with NIR fluorescence (Qin et al., 2017; Li et al., 2019). We expected that with the continuous improvement of CRISPR-Cas9-based imaging technology, important understandings can be obtained to help decipher the function of genes and the mechanisms of diseases.

## 2 The Mechanism of CRISPR-Cas9 System

**FIGURE 1:**
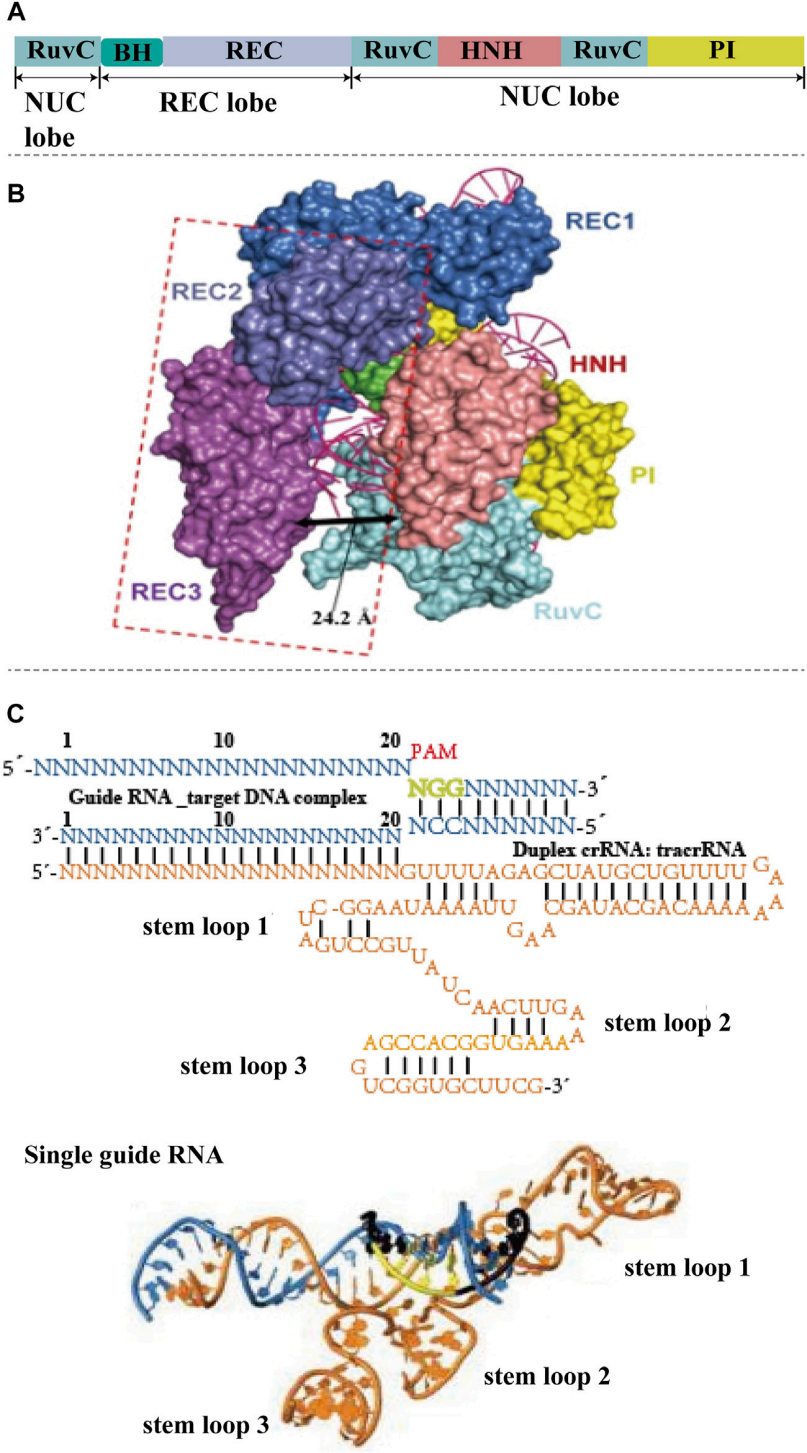
The structure of CRISPR-Cas9 system. **(A)** Basic structure domains of Cas9. Cas9 contains five different domains, RuvC domain, BH domain, REC domain, HNH domain, and PI domain, which are divided into nuclease (NUC) lobe and recognition (REC) lobe. **(B)** Crystal structures of CRISPR-Cas9. The HNH, RuvC, and PI domains reside in the Cas9 NUC lobe. The REC lobe of Cas9 contains other regions that interact with the sgRNA–DNA duplex. The HNH nuclease domain is responsible for slicing the complementary DNA strand of sgRNA, while the RuvC domain is responsible for slicing the other strand. Domains with conformational changes are indicated by dashed lines (reproduced from (Guo et al., 2019) with permission from Cell research). **(C)** Schematic of the sgRNA–target DNA complex-(N-any nucleotide), and three-dimensional structure of sgRNA. The target DNA shows in blue, PAM is labeled by green. The orange color shows the sequence and structure of sgRNA, which contains three stem loops (reproduced from (Anders et al., 2014) with permission from Nature).

The CRISPR-Cas9 system consists of two components: A Cas protein with DNase properties (Cas9) and a single-stranded guide RNA (sgRNA) (Knott and Doudna, 2018; Li L. et al., 2018). Cas9 protein is an RNA-dependent endonuclease containing two nuclease domains, HNH domain and RuvC domain, which are responsible for cutting the complementary and non-complementary strands by pairing sgRNA with the targeting sequence, respectively. (Figures 1A,B) (Jiang and Doudna, 2017). sgRNA is formed by crRNA and tracrRNA (Figure 1C). TracrRNA facilitates the processing of crRNA to mature. The crRNA helps the CRISPR-associated complex to recognize a specific target region of the foreign DNA and guide Cas-proteins to cleave foreign nucleic acid. A 20-base length sequence in the 5′end of sgRNA can be recognized by Cas9 protein and guide to the characteristic target region. PAM (protein assistant motif), a number of nucleotides adjacent to the target site, is a unique and critical component of invading DNA, because CRISPR-Cas needs it to identify and destroy the foreign DNA to produce a double-strand break (DSB). 5′-NGG-3′ as functional PAM can be recognized by Cas9 (Liu et al., 2021). In addition to CRISPR-Cas9, two other Cas proteins have been studied extensively, Cpf1 (also known as Cas12a) and Cas13a. CRISPR-Cpf1 system is more convenient compared with CRISPR-Cas9 system (Zetsche et al., 2015). On one hand, Cpf1 has a lower molecular weight than Cas9, which makes it easier to enter cells and improves the editing rate. On the other hand, Cpf1 only requires crRNA to mediate cleavage, while Cas9 requires the assistance of crRNA and tracrRNA. The major characteristic of Cas13a is that it edits RNAs (Gootenberg et al., 2017). Once Cas13a recognizes and cleaves the target RNA specified by the crRNA, it switches into an enzymatically activated state to bind and cleave any RNAs, regardless of whether they are homologous to crRNA.

**FIGURE 2:**
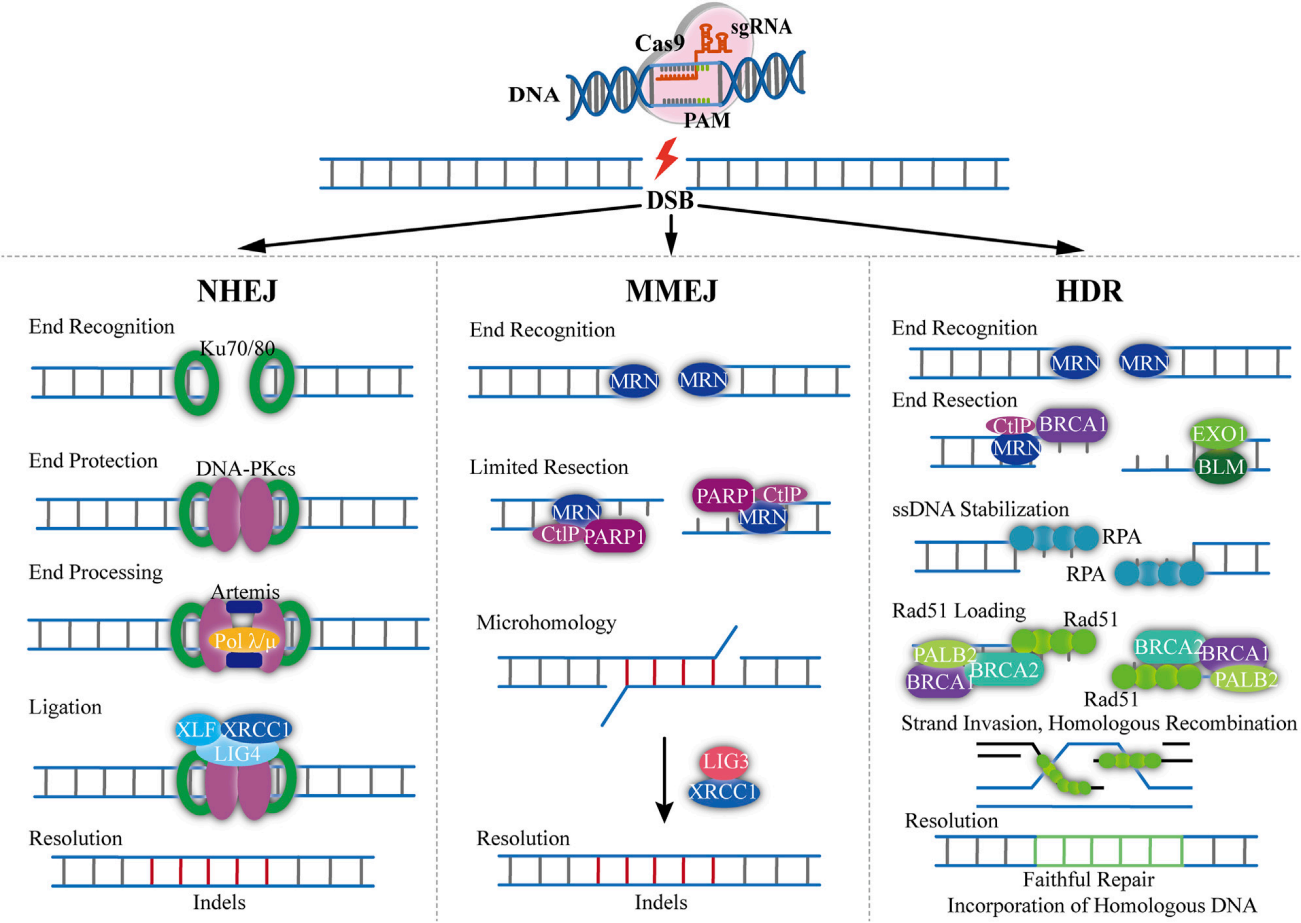
Overview of mechanism of CRISPR-Cas9-based genome editing. The Cas9/sgRNA can generate DSB resulting in exposed DNA ends that can be recognized by either Ku70/80 or MRN end-recognition complexes (NHEJ). When bond to Ku70/80, DNA repair proceeds through classical non-homologous end joining. The recruitment of DNA-PKcs, Artemis and DNA Polλ/µ coordinates end processing, resulting in small insertions and deletions less than 20 nucleotides. The ends are then ligated with the DNA LIG4, XRCC4, and XLF1 complex (MMEJ). DNA repair through the MMEJ pathway begins with the recognition of DNA ends by the MRN complex. The recruitment of PARP1 and CtIP results in limited 5′-end resection and exposure of 3′-ssDNA overhangs that can locally interact with regions of microhomology. Single-stranded DNA overhangs are created by annealing of complementary DNA strands and then removed, and the intervening gap in DNA sequence will be filled in by the activity of DNA Pol θ. XRCC1 and DNA LIG3 then contribute to the repair of DNA break (HDR). With sister chromatids, DNA repair can proceed through HDR pathway. The recognition of DSBs by the MRN complex initiates HDR and the recruitment of BRCA1 and CtIP facilitates limited 5′end resection followed by longer end resection by the BLM/EXO1 protein complex. The exposed ssDNA is initially bonded and stabilized by the RPA protein, which is subsequently replaced by RAD51 with the assistance of the BRCA1-BRCA2-PALB2 complex. The Rad51 nucleoprotein filaments mediate search and invasion on the homologous template, resulting in faithful incorporation of the homologous sequence with the broken DNA ends.

The Cas9 induced DSB is repaired under the mechanisms of non-homologous end joining (NHEJ), homology directed repair (HDR) or micro-mediated end joining (MMEJ) (Figure 2) (Zhang et al., 2017). The NHEJ pathway is most commonly used to create deletions and specific gene knock-out (Scully et al., 2019). It directly connects the end of the broken strands (Barman et al., 2020). During the NHEJ process, random insertion, substitution or deletion of bases will occur, which will cause gene mutations (Broeders et al., 2020). NHEJ can also cause frameshifts in the coding sequence of a gene to produce premature truncations, leading to an effective gene knock-out. The HDR pathway requires a homologous DNA sequence (donor DNA) (Nami et al., 2018). Typically, donor DNA for HDR is approximately 750–1,000 kb, which is homologous to that flanking the genomic cleavage site (Petolino, 2015). Homologous recombination is the desired mechanism for precise genome editing, which only happens in the presence of a homologous duplex template (Devkota, 2018). Hence, HDR can achieve precise base insertion or replacement by exogenously introducing homologous templates (Wang et al., 2017). However, the frequency of HDR appears to be extremely low. The MMEJ pathway is a way of DSB repair mediated by micro-homology (MH), which requires micro-homology sequences for repairing comparing with HDR (Bukhari and Muller, 2019). DSB exposed complementary sequences range from 5 to 25 nucleotides (microhomologies) (Seol et al., 2018). These microhomologies are used to align the DNA ends with the occurrence of end bridging. A polymerase then fills in any gaps, ultimately followed by ligation (Zinovkina, 2018). The mutant efficiency of MMEJ is similar to the NHEJ mechanism, which show a high potential for precise gene editing compared with HDR (Seol et al., 2018). Yao and co-workers showed that the mutation frequencies were as high as 20% by using MMEJ-mediated knock-in, which was approximately 10-fold higher than the HDR-based approach (Van Vu et al., 2021).

## 3 CRISPR-Cas9-Based Imaging System

### 3.1 Cas9-Based Imaging Systems

**FIGURE 3:**
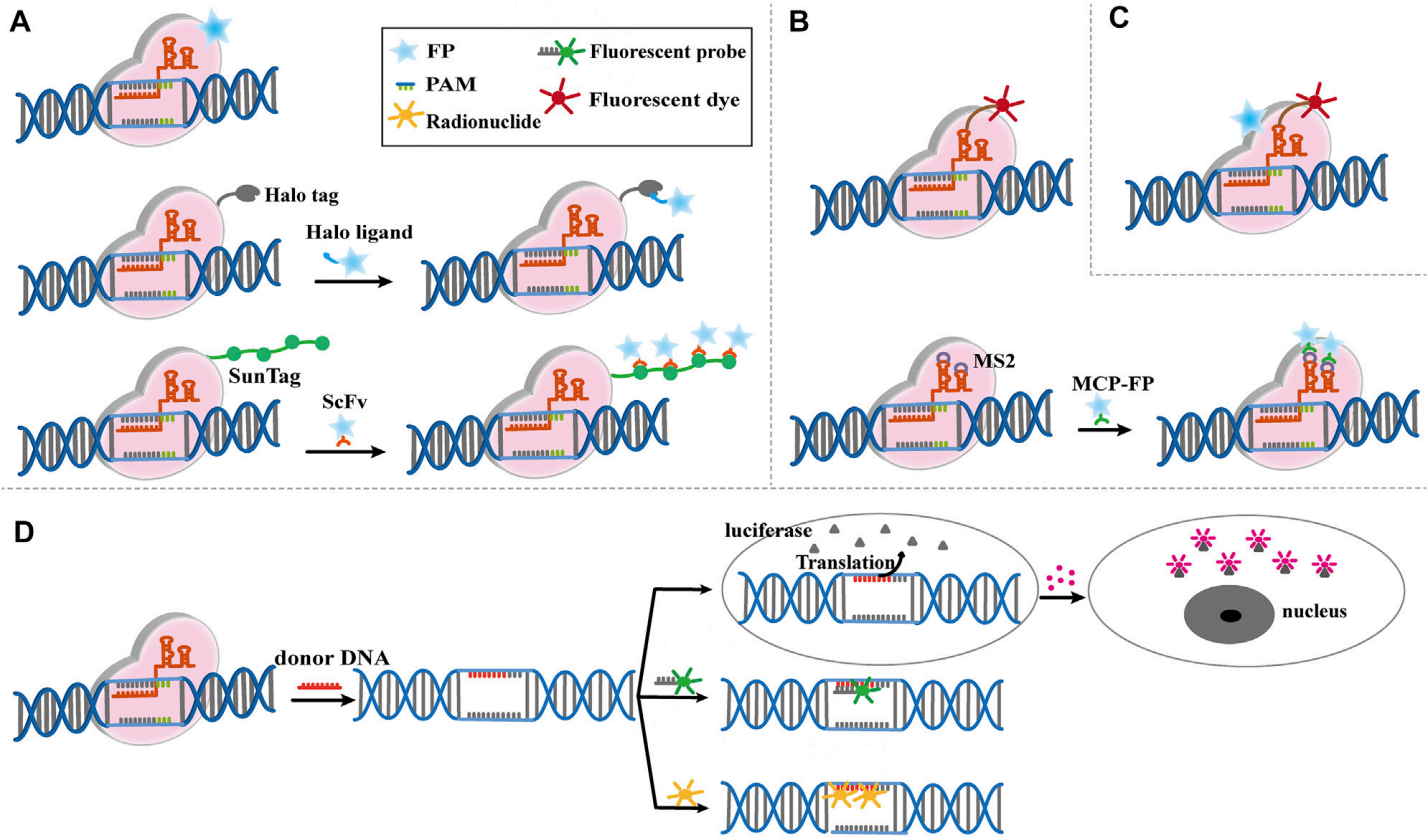
Modified CRISPR-Cas9-based imaging system. **(A)** Modified Cas9-based imaging systems, including Cas9 directly connect to fluorescent protein (FP), Halo Tag or Sunspot system. The upper part shows the FP fused Cas9 label and characterize the targeting DNA specifically mediated by sgRNA. The middle part shows the Cas9-Halo Tag system. Cas9 is labeled by Halo tag, which can find target DNA by sgRNA, then the FP fused Halo ligand recognize Halo-Cas9-sgRNA-DNA complex, and give the fluorescence to mark the DNA position. The bottom part shows the basic principle of CRISPR-Sunspot system, which is similar to Cas9-Halo Tag system. **(B)** Modified sgRNA-based imaging systems, including sgRNA directly connect to fluorescent dye or RNA aptamers. The upper part shows the fluorescent dye labed sgRNA is directly used to image target DNA and mediate Cas9 to edit genome. The bottom part shows the RCasFISH system for imaging. MS2 labled sgRNA bind with MCP-labeled fluorophores, and this complex target and characterize special DNA through Cas9. **(C)** Modified Cas9/sgRNA-based imaging systems. FP-labeled dCas9 and fluorescent dye labeled sgRNA complexes bind with the target DNA, which realize multiple color imaging. **(D)** Reporter gene imaging systems modified by CRISPR-Cas9. The reporter gene is inserted by CRISPR-Cas9 and bind to the fluorescent probe or radionuclide probe for imaging. Alternatively, the luciferase reporter gene is generated to bind to the substrate for imaging.

Living cell imaging could be achieved by fusing Cas9 with the fluorescent protein (GFP, eGFP or mCherry) to further label and characterize the targeting DNA (Figure 3A) (Chen et al., 2013; Maass et al., 2018). sgRNA-labeled targeting genes could be recognized by the Cas9-GFP to create imaging of living cells (Chen et al., 2018). However, this condition is limited by the high cost of oligo probes. Therefore, it is of great significance to develop a simpler, efficient and robust Cas9-based imaging systems. The CASFISH system, which comprised of dCas9 fused Halo and Halo ligands-conjugated fluorescent dyes, showed remarkably rapid imaging and was applicable for the detection of primary tissue sections (Figure 2A) (Deng et al., 2015). Similarly, the (Po)STAC system utilized scFv-dCas9 fusion protein and GFP-fused scFv antibody to produce fluorescence (Neguembor et al., 2018). By providing the PAM as part of an oligonucleotide (PAMmer), Ning-He Sun et al. (2020) developed a CRISPR-Sunspot system that allows efficient imaging of low-abundance mRNAs. Based on the SunTag system, dCas9 was linked to 24× GCNs, a polypeptide that can recruit specific proteins to achieve signal amplification, could recognized by scFv-GFP proteins, thus the signal was amplified. CRISPR-Sunspot was used to track co-localization of *Camk2a* mRNA with its regulatory protein *Xlr3b* in neurons, which provided a novel strategy to unravel the molecular mechanisms of diseases caused by aberrant mRNA molecules. dCas13b-eGFP fused with sgRNA can also be used to track and study the dynamics of nuclear domain-related lncRNAs (Yang et al., 2019).

### 3.2 sgRNA-Based Imaging Systems

In addition to use dCas9 fused fluorescent proteins, modified sgRNA can also be performed for imaging. A fluorophore-based imaging system consisting of dCas9 and molecular beacon (MB) linked sgRNA had been developed for live-cell visualization (Figure 3B) (Wu et al., 2018). Hong et al. (2018) developed a CRISPR-dCas9 system with bimolecular fluorescence complementation, which used the SunTag system to recruit the N-terminal portion of Venus fluorescent protein and the RNA aptamer (MS2) to recruit the C-terminal portion of Venus fluorescent protein. They were co-transfected with SunTag-dCas9 and MS2-gRNAs for telomere and single genomic locus labeling. Only co-transfection of these two systems could form a complete glowing Venus protein, which solved the false signal interference caused by the accumulation of gRNA. Cell imaging could be achieved by modifying sgRNA with RNA aptamers, which can recruit fluorescent labeled RNA-binding proteins for imaging. The RCasFISH system, which contained sgRNA-linked MS2 and MCP-labeled fluorophores, could be used for *in situ* quantification of RNA transcriptase in paraffin-embedded tissue (Figure 3B) (Wang et al., 2020). Similarly, this system could perform by lncRNA-protein complex for live cell imaging (Wang et al., 2019; Chen B. et al., 2020). When multiple RNA aptamers fused with different sgRNAs, and different fluorescent labeled RNA-binding protein could be recruited to achieve multicolor imaging (Ma et al., 2016; Wang et al., 2016; Ma et al., 2018; Maass et al., 2018). This method was expected to be a powerful tool for studying the dynamic intrachromosomal and inter-chromosomal interactions during cell cycle progression, while multiple fusion proteins complicate the process of building cell lines.

### 3.3 Multicolor Imaging Systems Based on Cas9/sgRNA

Multicolor imaging could also be achieved by modifying Cas9 and sgRNA simultaneously (Figure 3C). Halo tag-labeled dCas9 and Cy3-labeled sgRNA complexes bind their target DNA with high affinity, allowing sequential or simultaneous probing of multiple targets and multicolor labeling of target loci in cells (Deng et al., 2015). Currently, the Cas9/sgRNA-based imaging systems have been used to measure the nuclear dynamics and the on-target residence time of dCas9-sgRNA complexes in living cells. Guan et al. (2017) also reported a multiplexed live imaging via CRISPR-Cas9 system. Genomic loci in cells were identified by dCas9-eGFP and modified sgRNA, which could recruit fluorescence-fused RNA-binding proteins. dCas9-eGFP protein and Cy3-labeled sgRNA as fluorescent ribonucleoproteins could visualize the genomic DNA in human bone osteosarcoma cell (Wang et al., 2019).

### 3.4 Reporter Gene Imaging Systems Driven by CRISPR-Cas9

Reporter genes are widely used to detect the distribution, content, and dynamic activity of proteins in tissues/organs. CRISPR-Cas9 system can be used to generate reporters for living cell imaging and molecular processes (Muntean et al., 2018; Yang et al., 2019; Peng et al., 2020). For example, the luciferase reporter gene was inserted under the promoter by donor DNA and sgRNA, and luciferin was recruited to produce bioluminescence imaging (Figure 3D) (Li Z. et al., 2018). An efficient and scalable system combining CRISPR-Cas9 with fluorescent repressor-operator system, named SHACKTeR, had been reported (Figure 3D). Tet operators (TetO) were inserted as a tag, and eGFP-fused Tet repressors (TetR) were used for visualizing the tag. This system successfully labeled the colon cancer cells (Tasan et al., 2018). Moreover, the combination of PET and CRISPR-Cas9 had been applied in clinical translation of cell-based therapeutics (Ostrominski et al., 2020). *HSVtk* gene was integrated into the *AAVS1* locus of human urinary-induced pluripotent stem cell-derived cardiomyocytes (hUiCMs) using CRISPR-Cas9 system. By combining the probe ^18^F-FHBG, which could be used for *HSVtk* tracking, PET imaging provided the insight into the fate of hUiCMs after transplantation (Figure 3D). In another study, *PSMA* was encoded into human thyroid carcinoma cells by CRISPR-Cas9 and tracked by ACUPA-Cy3-BF3, a small-molecule that delivers positron-emitting fluoride (^18^F) and a fluorophore (Cy3) to report PSMA expression (Guo et al., 2019). PSMA is an attractive target for the diagnosis and treatment of prostate cancer patients, thus this reporter system showed great potential in PET/fluorescence-guided radical prostatectomy.

## 4 NIR Imaging and NIR/CRISPR-Cas9-Based Imaging Systems

### 4.1 NIR Directed Delivery and Activation of CRISPR-Cas9 System

**FIGURE 4:**
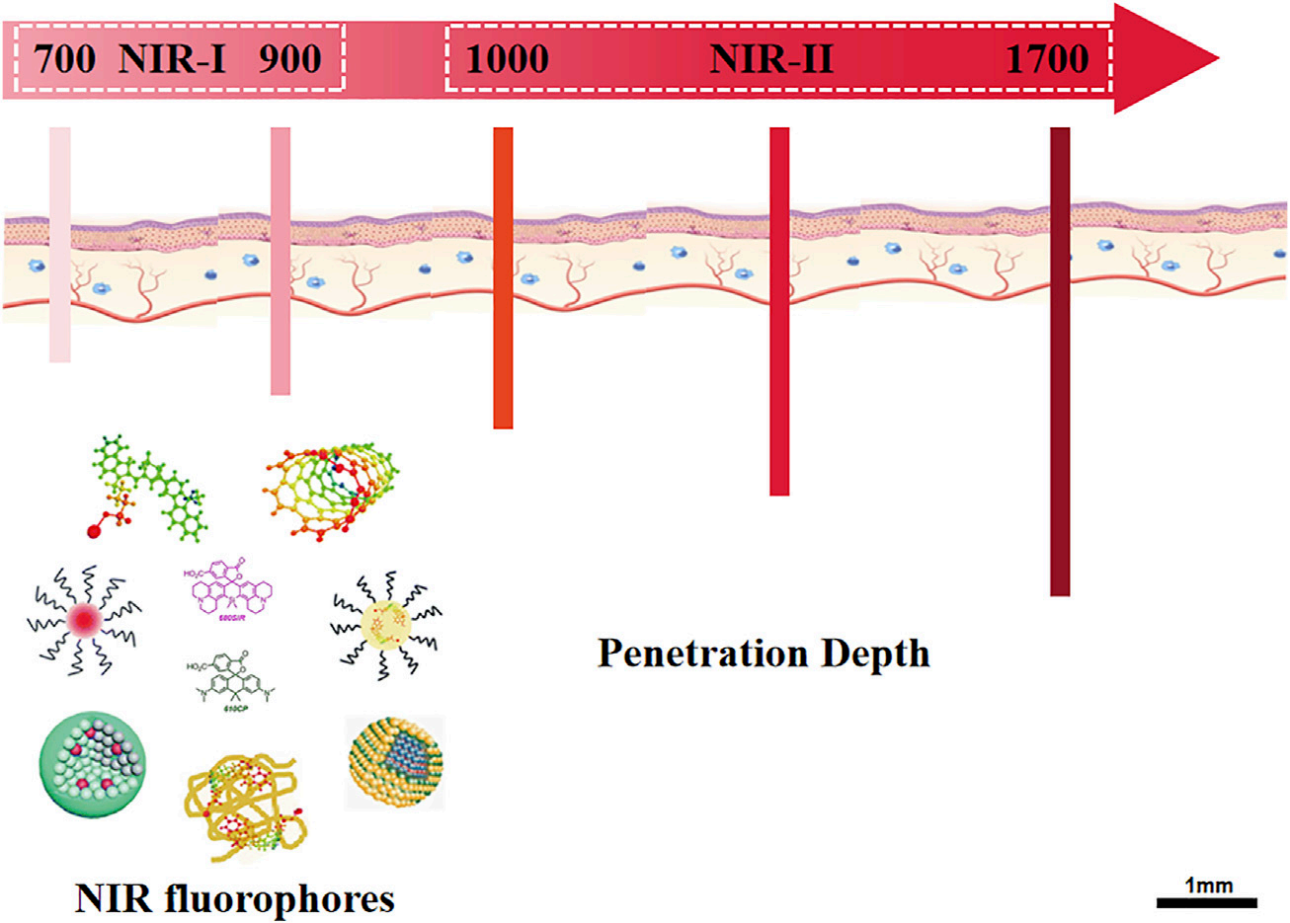
Overview of NIR fluorescence imaging. The schematic illustrated the sub-regions of NIR fluorescence, NIR-I (700–900 nm) and NIR-II (1,000–1700 nm). After excitation, the NIR fluorophores emit fluorescence with penetration depth of 3.0–4.3 mm (NIR-I) and 5–10 mm (NIR-II), respectively. NIR-II imaging shows superiority of low scattering and reflection of the fluorescence as well as low interference of background fluorescence.

Near-infrared (NIR) fluorescence imaging is mainly based on Near-infrared I (NIR-I, 700–900 nm) and Near-infrared II (NIR-II, 1,000–1,700 nm) (Figure 4) (He et al., 2018). Compared with visible light, biological tissues absorb and scatter less light in the near-infrared band. So NIR fluorescence imaging has significant advantages in providing physiological and pathological information with less damage to biological tissues and less interference to background fluorescence (Li et al., 2020a; Huang and Pu, 2020).

Programmable and inducible genome editing can be manipulated through optical regulation in the NIR window which include photothermal or photosensitive. For photothermal induced CRISPR-Cas9 release, the nanosystem is composed of a photothermal converter and the Cas9 plasmid driven by a heat-inducible promoter. When the plasmid was delivered to the targeted cells, the photothermal converter transform the harvested external light into intracellular local heat. The local heat induced the activation of heat-inducible promoter, thus results in the transcription of Cas9/sgRNA. However, once the light irradiation is switched off, the decreased temperature loss the capacity of intiating the transcription process. For photosensitive induced CRISPR-Cas9 release, the nanosystem can readily undergo photoisomerization or the chemical bond cleavage to release CRISPR-Cas9 when exposed to NIR light.

#### 4.1.1 Delivery and Activation of CRISPR-Cas9 in NIR-I Region

Better tissue differentiation is progressively afforded using fluorescent constructs in the NIR-I spectral region when compared to those emitting in the visible spectral region. The use of NIR-I fluorescent probe can accurately delineate the contour of superficial tumor, and guide the removal of tumor tissue during the operation (Croce and Bottiroli, 2014). However, NIR-I imaging suffers from shallow imaging depth, low contrast, and poor clarity caused by light scattering and autofluorescence. The penetration depth is still not enough for solid tumors due to high tissue scattering.

**FIGURE 5:**
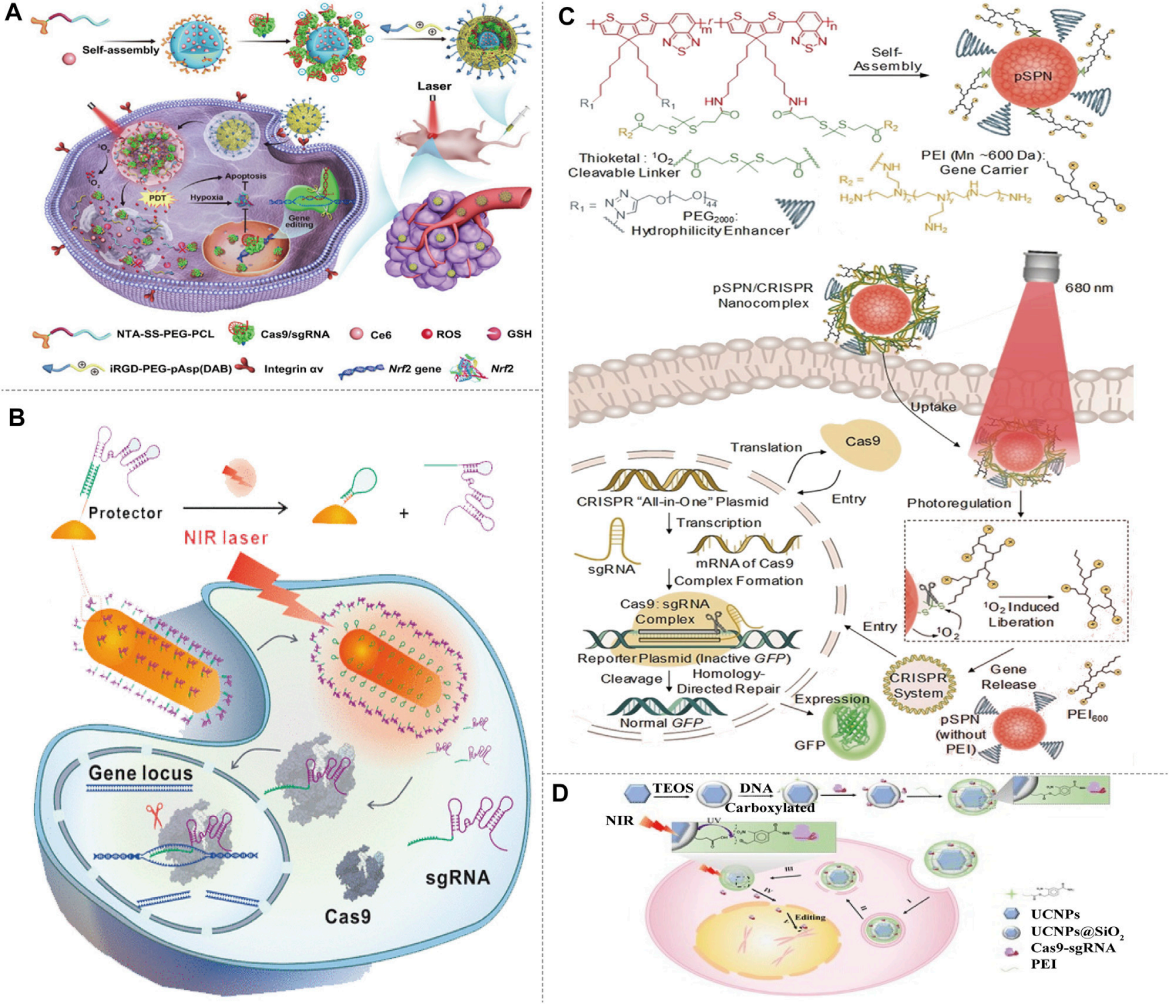
Light-activated NIR-I/CRISPR-Cas9 genome-editing strategy. **(A)** Schematic of the preparation, delivery, and intracellular fate of NIR-sensitive and reducing agent sensitive nanoparticles containing Nrf2-targeting Cas9/sgRNA and the antitumor photosensitizer Ce6. The self-assembly anionic micelles encapsulating Ce6 bind with His-tagged Cas9/sgRNA, and coat with cationic iRGD-modified copolymer to form nanoparticles, then the nanoparticles target and enter into tumor cells via integrin-iRGD binding. Ce6 generates ROS with the NIR irradiation, and Cas9/sgRNA is released to target the antioxidant gene *Nrf2*, enhancing tumor cell sensitivity to ROS synergistically with Ce6 (reproduced from (Chen et al., 2021) with permission from Science Advances). **(B)** Delivery and intracellular activation of a CRISPR-Cas9 genome-editing nanomachine, activated by a near-infrared laser. A sgRNA is hybridized to a protector DNA that is conjugated on a gold nanorod. NIR irradiation generates heat and results the release of the sgRNA. After sgRNA release, the protector forms a hairpin structure to hinder sgRNA rehybridization. The sgRNA and Cas9 protein then initiate gene editing (reproduced from (Tao et al., 2021) with permission from ACS Nano). **(C)** Illustration of NIR-I photolabile semiconducting polymer nanotransducer (pSPN)-mediated delivery and photoregulation of CRISPR-Cas9 gene editing. pSPN is self-assembled from a polymer whose backbone is grafted with poly (ethylene glycol) (PEG_2000_) and polyethylenimine (PEI_600_) brushes. The polymer backbone is able to generate singlet oxygen under NIR light irradiation, and PEI brushes act as cleavable gene carrier. pSPN can electrostatically bind the CRISPR and the GFP reporter plasmids to form nanocomplexes, and enabling noninvasive remote regulation of CRISPR/Cas9 gene editing using NIR light (reproduced from (Lyu et al., 2019) with permission from Angewandte Chemie (International ed. in English)). **(D)** Design of the UCNP-based CRISPR-Cas9 delivery system for NIR light-controlled gene editing. CRISPR-Cas9 was covalently anchored on UCNPs by photocleavable molecules and then coated with PEI to assist endosomal escape. NIR-triggered the cleavage of Cas9-sgRNA from UCNPs and initiate genome editing (reproduced from (Pan et al., 2019) with permission from Science Advances).

NIR-I probes have been used for CRISPR-Cas9 delivery and gene therapy. NIR light-triggered thermo-responsive copper sulfide was reported to serve as a “photothermal converter” and stably convert NIR-I light into local thermal effect to release CRISPR-Cas9 (Chen et al., 2021). The codelivery of CRISPR-Cas9 and photosensitizer chlorin e6 (Ce6) can be used in spatial gene editing. Upon NIR irradiation, Ce6 generates reactive oxygen species (ROS), lysosomal escape of nanoparticles was triggered, and Cas9/sgRNA was released in cytoplasm to achieve genome editing (Figure 5A) (Deng et al., 2020). Tao et al. (2021) developed a nanoplatform based on AuNCs, which could deliver Cas9/sgRNA plasmids to cancer cells and released them to achieve efficient genome editing. Peng et al. (2020) reported a NIR-I laser-activated CRISPR-Cas9 nanomachine (LACM). The LACM was irradiated by NIR-I laser to generate heat for sgRNA releasing, which guided the CRISPR-Cas9 genome editing, successfully knocked out the *PLK1* gene and induced apoptosis of the target cells (Figure 5B). Combining NIR-I with the photosensitive or light-to-heat conversion elements can spatiotemporally regulate CRISPR-Cas9-based gene editing. Lyu et al. (2019) developed a photolabile semiconducting polymer nanoparticles with NIR-I photoirradiation, which can spontaneously trigger the cleavage of CRISPR-Cas9 by nanoparticles, resulting in the release of CRISPR-Cas9 and subsequent initiation gene editing (Figure 5C). Upconversion nanoparticles which can convert NIR-I light into local ultraviolet light for the cleavage of photosensitive molecules, resulted in on-demand release of CRISPR-Cas9 (Figure 5D). By targeting the tumor gene *PLK1*, the proliferation of tumor cell can be successfully inhibited via NIR light activated gene editing (Pan et al., 2019). In addition, the combination of CRISPR-Cas9 and upconversion nanoparticles provided the possibility for remote DNA methylation (Chi et al., 2021).

#### 4.1.2 Delivery and Activation of CRISPR-Cas9 in NIR-II Region

Due to the reduction of background autofluorescence from biomolecules, photons at longer wavelengths provide higher contrast between the objects of interest and the background and penetrate deeper into living tissue. Biomedical imaging in NIR-II window can fully improve the temporal and spatial resolution of imaging (about 50 ms and about 25 µm) and penetration depth (up to 10 mm), obtain better image quality and signal-to-background ratio than NIR-I (He et al., 2018). NIR-II fluorescence imaging has been widely used in tumor imaging, liver imaging, small blood vessel imaging, lymph node imaging, and non-invasive dynamic cerebrovascular imaging over the past years (Hong et al., 2014; Zhao et al., 2019; Su et al., 2021). NIR-II imaging can discriminate tumor lesions more effectively than NIR-I imaging and can visualize lesions that are missed in NIR-I imaging (Zhang et al., 2021). A targeted activatable fluorescent nanoprobe in the NIR-II range was reported for *in vivo* optical dynamic imaging of traumatic brain injury (Li et al., 2020b).

Compared with NIR-I imaging, *in vivo* NIR-II fluorescence imaging is a relatively newer field of research. Nanomaterials display intrinsic fluorescence emission in the NIR-II window, allowing modulation of emission wavelengths well past the 1,000-nm mark. With the continuous development of chemical synthesis, new fluorophores are constantly discovered, including organic and inorganic fluorescent probes. Organic dyes are the earliest and most researched fluorophores and have been widely used in NIR-II imaging (Li and Pu, 2019; Li et al., 2021). While inorganic fluorescent probes have longer fluorescence lifetime, higher quantum efficiency, and higher fluorescence intensity (He et al., 2019; Nicholas et al., 2019; Yang et al., 2020).

**FIGURE 6:**
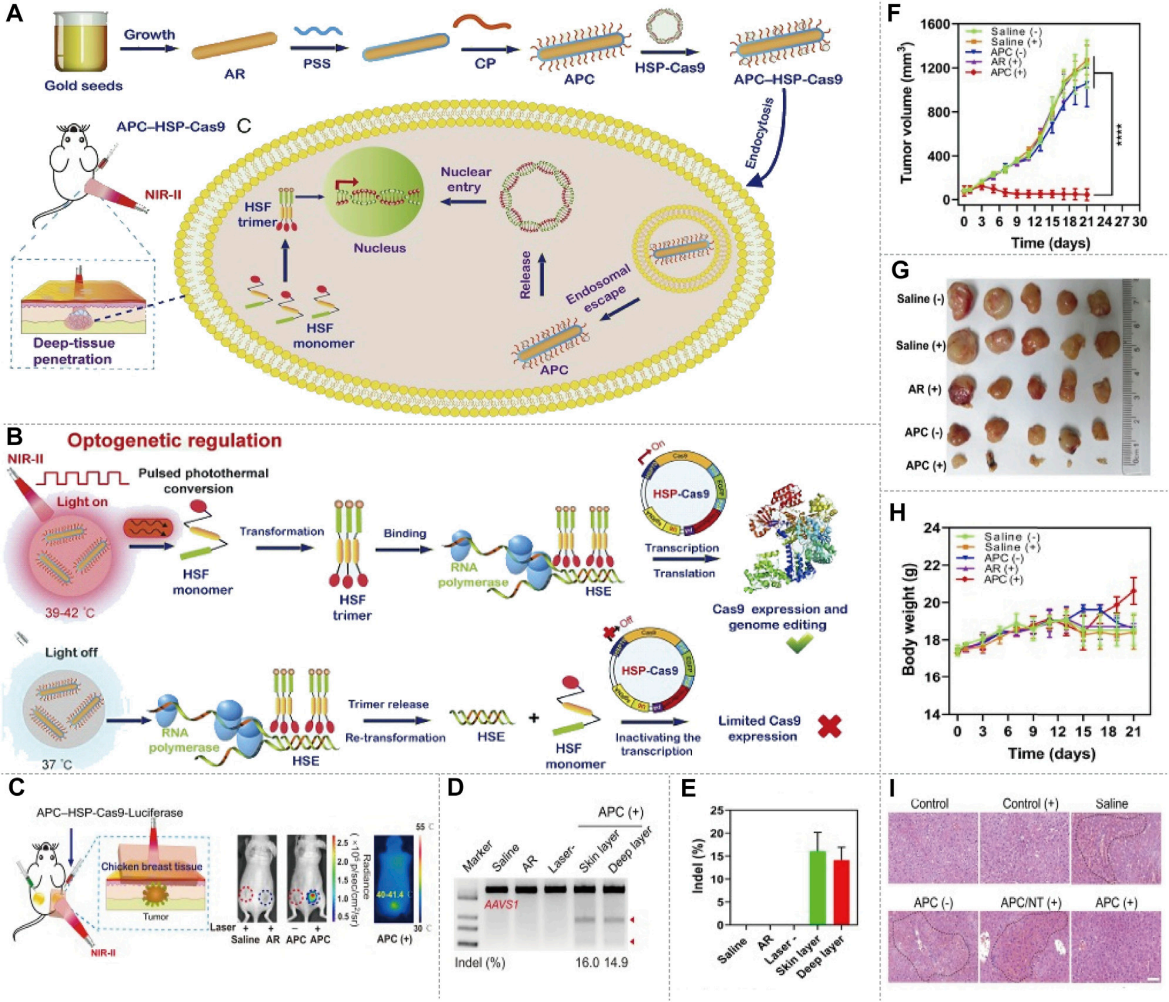
NIR-II/CRISPR-Cas9 system optogenetic regulation of genome editing. **(A)** The schematic of preparation of the APC–HSP complex, delivery of APC–HSP-Cas9 complexes and deep-tissue penetration. The APC–HSP-Cas9 complex is composed of a cationic polymer-coated Au nanorod (APC) and the Cas9 plasmid driven by a heat-inducible HSP70 promoter. APC plasmid is internalized by the targeted cell through endocytosis. The Au nanorod serves as a photothermal transducer to transform the NIR light into intracellular local heat to trigger the transcription of Cas9 and sgRNA. **(B)** The mechanism of inducible optogenetic regulation of Cas9-mediated genome editing. Upon NIR light irradiation, APC quickly generates localized heat, which induce the transformation of the heat-shock factor (HSF) from monomers to trimers. The HSF trimers bind with the heat-shock element (HSE) of the HSP70 promoter results in the transcription of Cas9. Once the light irradiation is switched off, the bound trimer is released from the HSE and back to monomers to inactivate the transcription process. **(C)** Tumor-bearing mice are administered APC–HSP-Cas9 complexes through peritumoral injection, and the tumor is then exposed to irradiation for 30 min in the presence of breast chicken tissue (5-mm thickness) covering the tumor position to simulate the deep-tissue condition, the luciferase expression shows optogenetic genome editing could be manipulated in the deep tissue of local lesions. **(D)** Indel mutations detected by T7E1 assay. A significant mutation is detected from both the surface and deep layer of the tumor tissues. **(E)** Quantitative analysis of indel mutations. The indel rate of the surface and deep layer of the tumor tissues is 16.0 and 14.9%, respectively. **(F)** Tumor growth curve after the transfection of APC–HSP-Cas9 complexes, followed by NIR light irradiation. The tumorbearing mice injected with APC HSP-Cas9 targeting *Plk1* exhibit significant tumor regression under irradiation. **(G)** Images of tumor tissues with different treatments. **(H)** The body-weight change during the treatment. A slight increase in body weight is observed at the end of the APC HSP-Cas9 targeting *Plk1* after irradiation treatment. **(I)** H&E staining of liver slices from mice 10 days after the treatment. The mice treated with galactose-modified APC HSP-Cas9 Fas significantly reduce hyperemia, shows the APC treatment merely induce any liver toxicity (reproduced from (Chen X. et al., 2020) with permission from Proceedings of the National Academy of Sciences of the United States of America).

**FIGURE 7:**
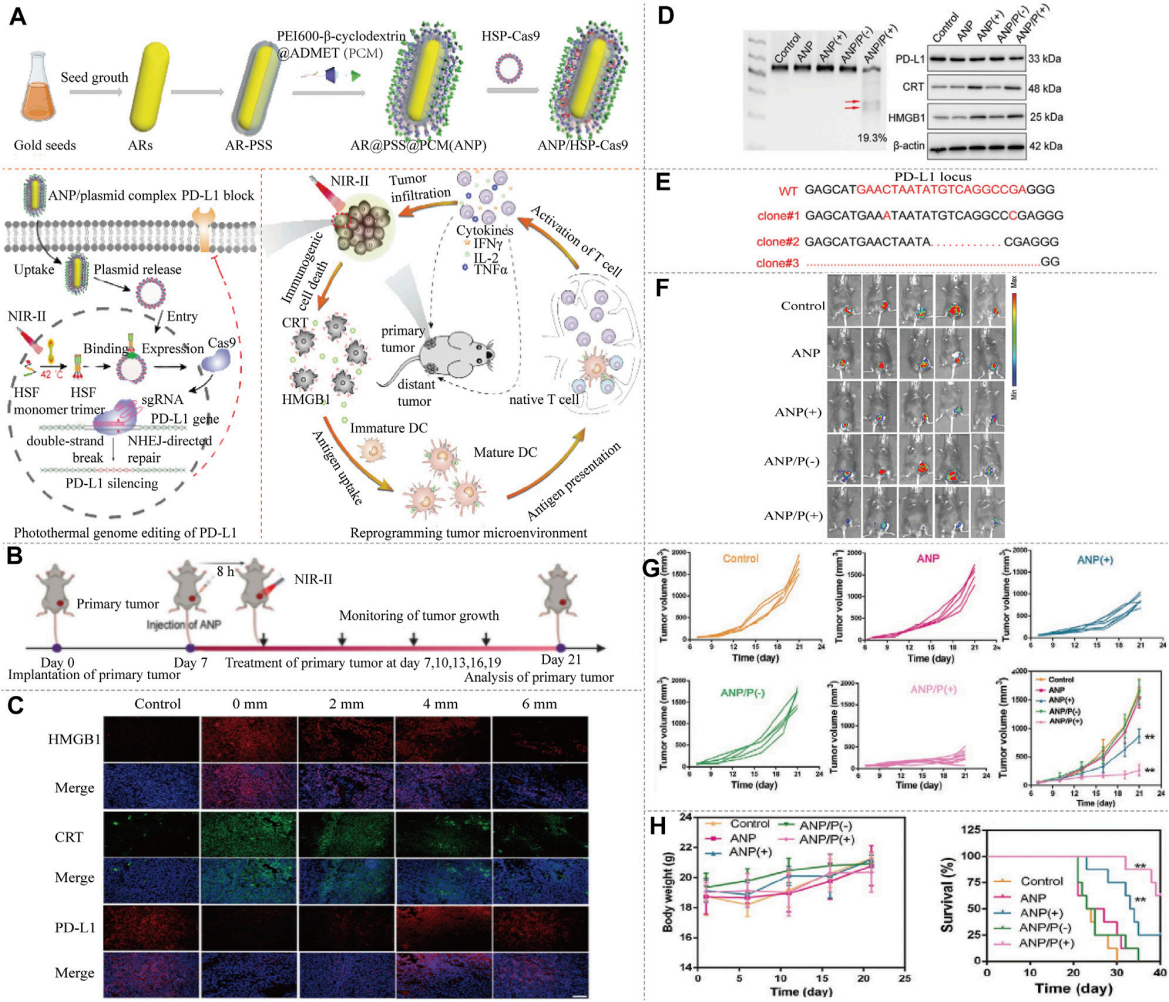
NIR-II/CRISPR-Cas9 system photothermal genome-editing strategy for cancer immunotherapy. **(A)** The frame of ANP/HSP-Cas9 complex synthesis, photothermal activation for PD-L1 genome editing and how to change the immunosuppressive tumor environment. ANR is composed of Au nanorods, biocompatible polystyrene sulfonate (PSS), and a supramolecular polymer (termed as PCM). Au nanorods act as a photothermal converter to induce unactive HSF monomer to active HSF trimer. The efficient intracellular delivery of ANP/HSP-Cas9 plasmid, can induce the transcriptional activation of Cas9 and sgRNA targeting PD-L1 upon NIR-II light irradiation, thereby enabling the precise genome editing of PD-L1. The heat produced by irradiation provide a mild condition which promotes immune memory, activates T cells, and improves T cell infiltration, transforms immunogenic-cold tumors into immunogenic-hot ones. This reprogramming tumor environment is beneficial for killing other distant tumor. **(B)** The schedule of tumor therapy by NIRII-controlled ANP/HSP-Cas9 *in vivo*. **(C)** Immunofluorescence analysis of HMGB1, CRT and PD-L1 protein expression from tumor tissue at different tumor depth. The significant CRT exposure within tumors can be implemented at depths up to 6 mm under NIR-II laser irradiation. **(D)** T7E1 assay was carried out to indicate indel mutations of PD-L1 in the tumor tissue. A high degree of genome editing was detected in tumor tissues. Western blot analysis of HMGB1, CRT, and PD-L1 proteins expression after different treated. The PD-L1 expression in tumors is significantly decreased by ANP-mediated genome editing. **(E)** Sanger sequencing results at PD-L1 locus from tumor tissue. Significant deletions and insertions are detected at the targeted loci around the PAM. ANP(+), ANP with NIR-II laser irradiation. ANP(−), ANP without NIR-II laser irradiation. ANP/P(+), ANP/HSP-Cas9 with NIR-II laser irradiation. ANP/P(−), ANP/HSP-Cas9 without NIR-II laser irradiation. **(F)** Representative *in vivo* bioluminescence images of mice after different treatment at day 21. **(G)** Tumor growth curves after being treated by ANP/HSP-Cas9 complexes with or without irradiation. **(H)** Body weight and Kaplan–Meier survival curves of tumor-bearing mice. ANP(+) significantly inhibit tumor growth, while the mice treated with ANP/HSP-Cas9 are more potent in delaying tumor growth after NIR-II laser irradiation. ANP(−) and ANP/P(−) show similar trend of tumor growth in comparison with the control group (reproduced from (Tang et al., 2021) with permission from Advanced Materials (Deerfield Beach, Fla.)).

Delivery of CRISPR-Cas9 applying NIR-II fluorophores has been reported. Light-to-heat conversion elements excited by NIR-II can transmit and regulate the release of CRISPR-Cas9. Chen X. et al. (2020) designed a nanosystem (termed nanoCRISPR), which comprised cationic polymer-coated Au nanorod and a heat-inducible Cas9 (Figure 6A). Au nanorod not only serves as a carrier but also can convert photonic energy into local heat to induce the expression of Cas9 endonuclease. Once NIR-II irradiation is switched off, the photothermal effects disappear, causing the inactivation of CRISPR-Cas9 transcription process (Figure 6B). Thus, gene editing can be easily and precisely controlled by fine-tuning the intensity and duration of NIR-II irradiation at multiple time points *in vitro* and *in vivo*. The authors also demonstrated that this strategy can be extended to the treatment of deep tumor and fulminant hepatic failure (Figures 6C–I). Tang et al. (2021) also reported a cationic gold nanorod for delivering CRISPR-Cas9 and inducing the expression of Cas9 (Figure 7A). It improved immune checkpoint blockade therapy by CRISPR-Cas9-mediated disruption of *PD-L1* and mild-hyperthermia-induced activation of immunogenic cell death. Gold nanorod converts NIR-II light into mild hyperthermia to induce both immunogenic cell death and the expression of Cas9. The genomic disruption of *PD-L1* significantly augments immune checkpoint blockade therapy by improving the conversion of dendritic cells to T cells, thereby reprogramming immunosuppressive tumor microenvironment into immunoactive one (Figures 7B–H). The optical regulation of CRISPR-Cas9 by NIR-II light imparts excellent spatial specificity and deep tissue penetration, this strategy therefore can precisely target tumor tissues and circumvent the immune-related adverse events. Moreover, NIR-II spectroscopy was sensitive to changes in chemical bonds of DNA caused by modifying genotype, which has been used in identifying CRISPR-Cas9-based mutants in rice, helping to accelerate the selection and crop breeding process (Feng et al., 2017).

### 4.2 NIR/CRISPR-Cas9-Based Imaging Systems

In Table 1, we summarize all the NIR fluorophores related to CRISPR-Cas9. Most NIR fluorophores were used for CRISPR-Cas9 delivery, but the NIR/CRISPR-Cas9-based imaging systems have not been widely studied. The NIR fluorescent imaging showed a deep penetration and high resolution in biological tissues, while the Cas9 and sgRNA can be modified to give imaging *in vivo*. Therefore, the combined application of NIR fluorophores and CRISPR-Cas9 has great innovation and potential in multicolor imaging.

**TABLE 1:** Near-infrared-I/II fluorophores related to CRISPR-Cas9.

NIR-I/II fluorophore	*Nanoparticles structures*	Excitation (nm)	Emission (nm)	Character	Targets	Application	Ref
SPPF-Dex nps	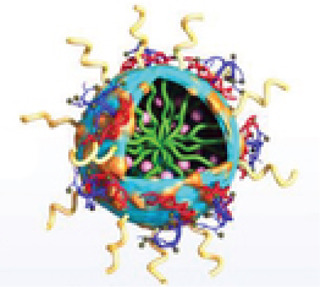	808	1,000–1,400	Photothermal conversion, NIR-II imaging, deep tissues	*MTH1*, HCT 116 cells	CRISPR-Cas9 delivery, real time imaging, animal model	Li et al. (2019)
Au nanorod (APC)	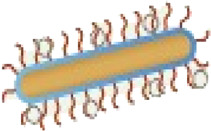	1,064	1,000–1,700	Photothermal activation, deep tissues	*AAVS1*, asialoglycoprotein receptors targeting, Liver targeting	CRISPR-Cas9 delivery, tumor therapy, animal model	Chen X. et al. (2020)
AR@PSS@PCM (ANP)	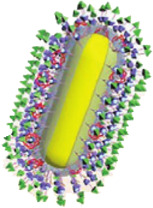	1,064	—	Photothermal activation, deep tissues	*PD-L1* targeting, ICD activation	CRISPR-Cas9 delivery, improving immune checkpoint blockade, animal model	Tang et al. (2021)
Protamine–AuNCs	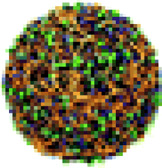	700–1,000	—	NIR-I imaging, deep tissues	*E7*, U2OS, HeLa cells	CRISPR-Cas9 delivery, tumor therapy, *in vivo*	Tao et al. (2021)
Photolabile semiconducting polymer nanotransducer	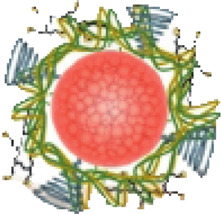	680/808	700–1,000	Photothermal activation, deep tissues	Plk1, Hela cells	CRISPR-Cas9 delivery, animal model	Lyu et al. (2019)
NTA-SS-PEG-PCL/Ce6 Complex	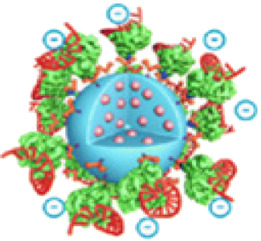	1,064	700–1,000	Photothermal conversion, deep tissues	*Nrf2*, CNE-2 cells	CRISPR-Cas9 delivery, tumor therapy, animal model	Deng et al. (2020)
UCNPs-Cas9@PEI	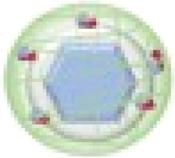	980	—	Photothermal activation, upconversion, deep tissues	Plk1, kB cells, A549 cells	CRISPR-Cas9 delivery, tumor therapy, animal model	Pan et al. (2019)
Upconversion nanoparticles	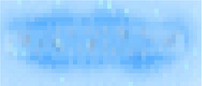	700–1,000	—	Photothermal activation, deep tissues	DNA 5-cytosine methylation, HEK293T cells	CRISPR-Cas9 delivery, animal model	Chi et al. (2021)
Gold nanorod (AuNR)	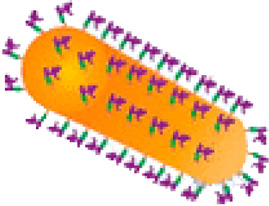	808	700–1,000	Photothermal therapy, deep tissues	*EGFP*, *EMX1*, A549, HEK293T cells	Gene editing, *in vivo*	Peng et al. (2020)
NIR light-triggered thermo-responsive copper sulfide (CuS)	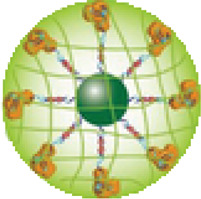	808	—	Photothermal therapy, deep tissues	*Hsp90α*, A375 cells	CRISPR-Cas9 delivery, tumor therapy, animal model	Chen et al. (2021)
Si-rhodamine 680SiR	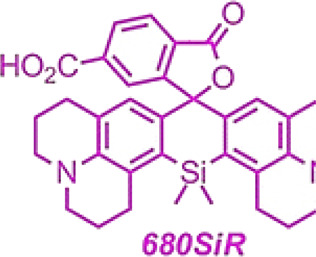 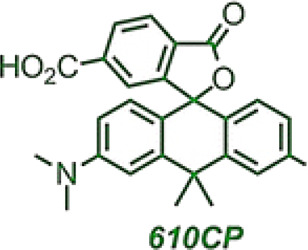	680	700	NIR-I imaging, deep tissues	SNAP targeting, tubulin2 targeting, U2OS cells	Dual-color confocal and STED imaging, *in vivo*	Butkevich et al. (2018)

**FIGURE 8:**
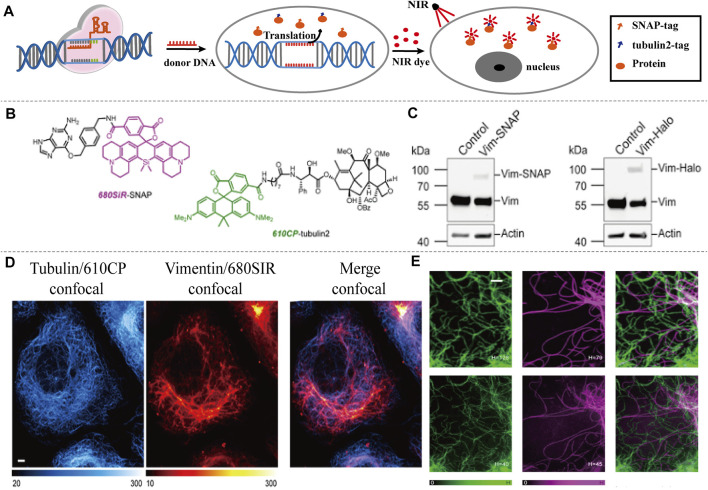
NIR-I/CRISPR-Cas9-based imaging systems. **(A)** CRISPR-mediated endogenous labeling of proteins could combine with NIR dyes, and show NIR imaging following by excitation. CRISPR-Cas9 is used to knockin SNAP-tagged or Halo-tagged protein from its genomic locus. NIR dye bind with SNAP-tagged or Halo-tagged protein to give image. **(B)** Structures of the probes used for two-color imaging. **(C)** Western blots of CRISPR-Cas9 mediated tagging of vimentin with SNAP Tag or Halo Tag show a higher expression. **(D)** Confocal images of vimentin-SNAP knock-in cells labeled with 610CP-tubulin2 and 680SiR-SNAP probes. **(E)** Upper row, confocal images of vimentin-SNAP stained with 680SiR-SNAP, microtubule cytoskeleton stained with 610CP-tubulin2, and merge. Lower row, the corresponding STED images (reproduced from (Butkevich et al., 2018) with permission from ACS chemical biology).

Butkevich et al. (2018) used CRISPR-Cas9 to generate and detect an endogenous tagged protein by combining with a newly established pair of NIR STED labels (Figure 8A). The synthesis of NIR fluorescent Si-Rhodamine Dye 680SiR was based on 8-Bromojulolidine 1. The 680SiR was expected for SNAP combination, and the other STED dye 610CP could be combined with cytoskeletal protein tubulin2 (Figure 8B). Then the U2OS cell lines expressing SNAP-tagged or Halo-tagged vimentin from its genomic locus were generated by the CRISPR approach. The transgene expression was verified by immunoblotting (Figure 8C). Both the 680SiR-SNAP and the 610CP-tubulin2 demonstrated high brightness and good imaging performance (Figure 8D). The cell-to-cell reproducibility of two-color staining was verified by confocal microscopy. U2OS cells with stable expression of vimentin-SNAP fusion proteins were incubated with 610CP-tubulin2 and 680SiR-SNAP, which showed a superior brightness imaging for clear color separation, and no observable cross-talk between two color channels was detected (Figures 8E,F). In this system, CRISPR-Cas9 offered a robust and reproducible label for NIR imaging *in vivo*. This approach of endogenous tagging of protein offered reliable and cell-to-cell reproducible dual-color nanoscale imaging in living cells.

**FIGURE 9:**
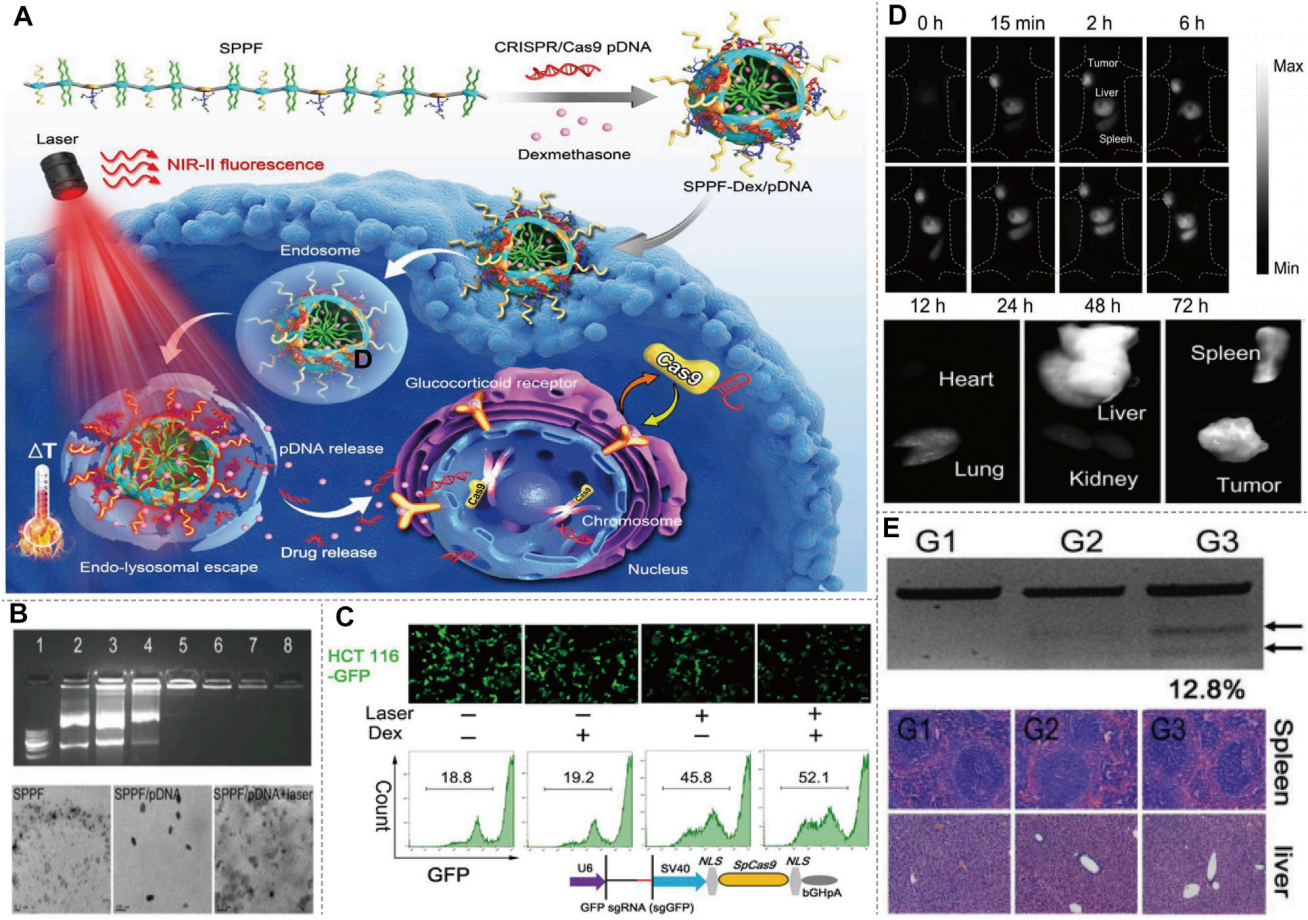
NIR-II/CRISPR-Cas9-based imaging systems. **(A)** Diagram of SPPF-Dex mediated CRISPR-Cas9 imaging, and illustration of the intracellular genome editing process upon 808 nm laser irradiation. Upon the NIR-II laser irradiation, the distribution of SPPF-Dex can be tracked *in vivo*. Simultaneously, the photothermal conversion of SPPF facilitates the endolysosomal escape of SPPF-Dex and release of CRISPR-Cas9. Dex dilates the nuclear pores to initiate the translocation of CRISPR-Cas9 for genome editing. **(B)** Agarose gel retardation assay of SPPF/pDNAs, morphologies of SPPF, SPPF/pDNA, and SPPF/pDNA after 808 nm laser irradiation detected by TEM showed high efficiency as a carrier. **(C)** GFP gene disruption efficacy after different treatment in engineered GFP labeled cells detected by confocal microscope and flow cytometry. **(D)** *In vivo* NIR-II imaging of the injection of SPPF-Dex/Cas9-GFP and *in vitro* NIR-II imaging of the tumor and other organs harvested to analysis of the *in vivo* distribution. **(E)** T7E1 assay was carried out to indicate indel mutations of PCR products of GFP gene retrieved from GFP labeled tumor with different treatment. Histological examination of H&E staining of spleen and liver sections showed a minimal side effect. G1, GFP labeled tumor treated with PBS and 808 nm laser irradiation. G2, GFP labeled tumor treated with SPPF-Dex/Cas9-sgGFP. G3, GFP labeled tumor treated with SPPF-Dex/Cas9-sgGFP and 808 nm laser irradiation (reproduced from (Li et al., 2019) with permission from Advanced materials (Deerfield Beach, Fla.)).

NIR fluorophores could not only be used to deliver and control the release of CRISPR-Cas9 through photothermal conversion, but also could be used to monitor the delivery path by NIR imaging. Li et al. (2019) described a type of rationally designed semiconducting polymers (SPs) brush and applied them to NIR-II imaging-guided light-triggered remote control of CRISPR-Cas9 genome editing. SPPF nanoparticles were fabricated by sequentially conjugating alkyl side chains, PEG chains, and fluorinated polyethylenimine (PF) to the backbone of the initial SPs (Figure 9A). The backbone of SPs served as the photothermal transducer, while the PF bound with CRISPR-Cas9 pDNAs via electrostatic interaction and supramolecular interaction. Dexamethasone (Dex) is used for encapsulating in the hydrophobic core of the formed SPPF nanoparticles. Approximately 30% HCT 116-GFP cells showed decreased GFP after SPPF/Cas9-sgGFP incubation upon laser irradiation, and Dex could further enhance the GFP editing efficiency (Figure 9C). The tumor tissue showed bright NIR-II fluorescence signal, suggesting that SPPF could be a good approach to monitor genome editing *in vivo*. NIR-II fluorescence signal were only observed in metabolically active organs, liver and spleen, indicating the biodegradability of SPPF (Figure 9D). SPPF-Dex/Cas9-sgGFP injection without laser irradiation, PBS or SPPF-Dex/Cas9-sgNull injection with laser irradiation, did not affect the GFP gene disruption (Figure 9E). No abnormal and inflammatory cell infiltration in spleen and liver was observed after treatment, suggesting SPPF exhibited good biocompatibility (Figure 9E). The innovation of this method is to perform visible and controllable *in vivo* genome editing under near-infrared light guidance and stimulation, which provides a paradigm for tracking the distribution of genome editing system in real time. More studies should be performed to develop this platform in CRISPR-Cas9-based therapeutic approach for genetic disease in the following work. The CRISPR-Cas9-based precise gene therapy and imaging will keep movement clinically in the near future.

## 5 The Delivery of CRISPR-Cas9-Based Imaging Systems

There are three types of the CRISPR delivery system: 1. delivery the plasmid DNA (pDNA) of Cas9 and sgRNA, 2. deliver the mRNA of Cas9 and sgRNA, 3 deliver the protein (ribonucleoprotein, RNP) of Cas9 and sgRNA, which depend on physical, biological, or chemical manner (Doetschman and Georgieva, 2017; Chen G. et al., 2019; Liu et al., 2019). Physical methods, such as electroporation, are most suitable for *in vitro* experiments. Viral vectors are commonly used for CRISPR-Cas9 delivery from single cell to *in vivo*. In clinical trials, more than 70% of gene drug carriers are also viruses (Kim et al., 2017). However, viral vectors may cause unnecessary immunogenicity and mutation risks in the host, thereby limiting their clinical transformation. In terms of biosafety, loading and encapsulation capability, non-viral vectors, especially nanocarriers, have broadened the application in CRISPR-Cas9-based gene therapy (Gu et al., 2018). Several nano-delivery systems for CRISPR-Cas9 have been developed, including cationic liposomes, lipid nanoparticles (LNPs), cationic polymers, vesicles, and gold nanoparticles (Gu et al., 2018). Although nanocarriers offer many advantages for CRISPR-Cas9 delivery in the treatment of cancer and other diseases, some problems remain to be solved. For example, nanoparticles are unstable in blood circulation, the biological distribution of nanoparticles is not ideal, low clearance rate and transfection efficiency, etc. Thus the most of nanocarriers used for CRISPR-Cas9 delivery stay in preclinical studies. More research on reforming nanomaterials of higher biocompatibility, higher efficiency and lower toxicity for loading CRISPR-Cas9 will need be developed. Table 2 shows the characteristic of different CRISPR-Cas9 delivery systems involved in CRISPR-Cas9-based imaging.

**TABLE 2:** The delivery system of CRISPR-Cas9-based imaging.

Delivery vehicle	CRISPR-Cas9 format	Character	Efficiency	Advantage	Disadvantage	Application	Ref
SPPF-Dex nps	Plasmid	Photothermal regulation	12.8% indel mutation rate in HCT 116-GFP cells	Low toxicity, endolysosomal escape, payloads release	Less loading	In cell, *in vivo*	Li et al. (2019)
Au nanorod (APC)	Plasmid	Photothermal regulation	18.0% indel mutation rate in Hepa1-6 cells, 6.7% indel mutation in the liver tissue	Specific binding	Surface modification is needed to bind genes effectively	In cell, *in vivo*	Chen X. et al. (2020)
AR@PSS@PCM(ANP)	Plasmid	Photothermal regulation	39.7% indel mutation rate in B16F10 cells, 21.5% in tumor	Good biocompatibility, promote intracellular delivery	prone to negatively charge cells and proteins	In cell, *in vivo*	Tang et al. (2021)
Protamine–AuNCs	Plasmid	Cationic protamine facilitates release	27.5% indel mutation rate in HeLa cells	Cell-penetrating properties, nucleus-targeting	Reunion	In cell	Tao et al. (2021)
Photolabile semiconducting polymer nanotransducer	Plasmid	Photosensitive regulation	Indel 15- and 1.8-fold enhancements in cells and living mice	Efficient release of gene vectors	Low particle surface potential	In cell, *in vivo*	Lyu et al. (2019)
NTA-SS-PEG-PCL/Ce6 Complex	Cas9 RNP	Photosensitive regulation	42.6% indel mutation rate in CNE-2 cells, 31.2% indel mutation rate in tumor	Stability, low toxicity, tumor targeting	Cytotoxicity	In cell, *in vivo*	Deng et al. (2020)
UCNPs-Cas9@PEI	Cas9 RNP	Photosensitive regulation	Inhibited cancer cell proliferation and tumor growth	Endosomal escape	Low particle surface potential, less loading	In cell, *in vivo*	Pan et al. (2019)
Upconversion nanoparticles	Plasmid	Photosensitive regulation	Inhibited cancer cell proliferation and tumor growth	Light stability, low potential toxicity, no background light interference	Low particle surface potential	In cell, *in vivo*	Chi et al. (2021)
Au nanoparticles	Plasmid	Photothermal regulation	1.24% indel mutation rate inA375 cells, sustainable tumor inhibition	High loading efficiency, good stability, good repetition	Surface modification is needed to bind genes effectively	In cell, *in vivo*	Wang et al. (2018)
NIR light-triggered thermo-responsive copper sulfide (CuS)	Cas9 RNP	Photothermal regulation	37.3%indel mutation rate in A375 cells, 23.8% indel mutationin in tumor	Assist endosomal escape	Reunion, cytotoxicity	In cell, *in vivo*	Chen et al. (2021)
Electroporation	RNP, plasmid	Transient transfection, easy to cause damage to cell membranes	High transfection efficiency	Small dependence on cell types, equipment cost	Easy to cause damage to cell membranes	In cell	Chen S. et al. (2019)
Viral vector (AAV, LV, AdV)	Plasmid	Has been approved for human clinical trials, cause virus contamination	Stable, high transfection efficiency	High capacity	Introduce virus contamination, immune response	In cell, *in vivo*	Moreno et al. (2020)
Lipofectamine	RNP, plasmid	Simple preparation, good safety, low cost	High transfection efficiency	Simple preparation, good safety	Prone to immune response	In cell, *in vivo*	Liu et al. (2020)

## 6 Conclusion

The application of the CRISPR-Cas9 system has gradually expanded from genome editing to real-time imaging of living cells. In this review, we described the composition and mechanism of CRISPR-Cas9 system, and summarized the research progress of CRISPR-Cas9-based imaging system in recent research. By modifying Cas9 protein, sgRNA or both with fluorophore, CRISPR-Cas9-based imaging systems achieve multi-color imaging and the combination of PET imaging. We mainly overviewed the NIR imaging and the studies of NIR/CRISPR-Cas9-based imaging systems. We also summarized the application of NIR fluorophores in the delivery of CRISPR-Cas9, which had been widely reported. But there are rarely attempts for combination NIR imaging with CRISPR-Cas9 system. Only two studies have reported NIR/CRISPR-Cas9-based imaging systems, one is for dual-color NIR imaging, and the other is for tracking the location of CRISPR-Cas9 system.

However, there are still some problems need to be solved in this field. Off-target effects have always been a major challenge to limit the application of Cas9 protein, the presence of off-target at high density may lead to false-positive locus detection, thus free fluorescence affects the signal-to-noise ratio and inaccuracy of imaging. More complicated strategies, such as fluorescence resonance energy transfer, can be used to eliminate free luminescence signals (Mao et al., 2019). Improving the FP signal or using a high sensitivity cutting-edge microscope can further optimize the signal-to-noise ratio and improve imaging efficiency (Lu et al., 2021). Delivery is another main challenge in CRISPR-Cas9-based imaging system (Glass et al., 2018; Qiu et al., 2019; Xu et al., 2021). The more efficient, higher compatibility, and lower biotoxicity delivery system will help to realize the clinical potential of this technology. Therefore, designing ideal carrier for targeting tissues of interest is the main task at this stage. It is believed that with the continuous deepening of research, the CRISPR-Cas9 system will be expected to exert its due imaging application value.

In order to successfully translate CRISPR-Cas9-based imaging in clinical applications, it is necessary to continuously perform human and animal experiments. Although many challenges need to be overcome, the potential of CRISPR-Cas9-based imaging will help solve many genome and chromatin mysteries. With the continuous improvement of the CRISPR-Cas9 application, it is expected to become an epoch-making biological technology in other fields.

## References

[B87] Anders C. Niewoehner O. Duerst A. Jinek M. (2014). Structural Basis of PAM-Dependent Target DNA Recognition by the Cas9 Endonuclease. Nature 513 (7519), 569–573. 10.1038/nature13579 25079318 PMC4176945

[B1] Barman A. Deb B. Chakraborty S. (2020). A Glance at Genome Editing with CRISPR-Cas9 Technology. Curr. Genet. 66 (3), 447–462. 10.1007/s00294-019-01040-3 31691023

[B2] Broeders M. Herrero-Hernandez P. Ernst M. P. T. van der Ploeg A. T. Pijnappel W. W. M. P. (2020). Sharpening the Molecular Scissors: Advances in Gene-Editing Technology. iScience 23 (1), 100789. 10.1016/j.isci.2019.100789 31901636 PMC6941877

[B3] Bukhari H. Müller T. (2019). Endogenous Fluorescence Tagging by CRISPR. Trends Cel Biol. 29 (11), 912–928. 10.1016/j.tcb.2019.08.004 31522960

[B4] Butkevich A. N. Ta H. Ratz M. Stoldt S. Jakobs S. Belov V. N. (2018). Two-Color 810 Nm STED Nanoscopy of Living Cells with Endogenous SNAP-Tagged Fusion Proteins. ACS Chem. Biol. 13 (2), 475–480. 10.1021/acschembio.7b00616 28933823

[B5] Chen B. Deng S. Ge T. Ye M. Yu J. Lin S. (2020). Live Cell Imaging and Proteomic Profiling of Endogenous NEAT1 lncRNA by CRISPR/Cas9-mediated Knock-In. Protein Cell 11 (9), 641–660. 10.1007/s13238-020-00706-w 32458346 PMC7452982

[B6] Chen B. Gilbert L. A. Cimini B. A. Schnitzbauer J. Zhang W. Li G.-W. (2013). Dynamic Imaging of Genomic Loci in Living Human Cells by an Optimized CRISPR/Cas System. Cell 155 (7), 1479–1491. 10.1016/j.cell.2013.12.001 24360272 PMC3918502

[B7] Chen B. Zou W. Xu H. Liang Y. Huang B. (2018). Efficient Labeling and Imaging of Protein-Coding Genes in Living Cells Using CRISPR-Tag. Nat. Commun. 9 (1), 5065. 10.1038/s41467-018-07498-y 30498221 PMC6265289

[B8] Chen C. Ma Y. Du S. Wu Y. Shen P. Yan T. (2021). Controlled CRISPR‐Cas9 Ribonucleoprotein Delivery for Sensitized Photothermal Therapy. Small 17 (33), 2101155. 10.1002/smll.202101155 34269521

[B9] Chen G. Abdeen A. A. Wang Y. Shahi P. K. Robertson S. Xie R. (2019). A Biodegradable Nanocapsule Delivers a Cas9 Ribonucleoprotein Complex for *In Vivo* Genome Editing. Nat. Nanotechnol. 14 (10), 974–980. 10.1038/s41565-019-0539-2 31501532 PMC6778035

[B10] Chen S. Sun S. Moonen D. Lee C. Lee A. Y.-F. Schaffer D. V. (2019). CRISPR-READI: Efficient Generation of Knockin Mice by CRISPR RNP Electroporation and AAV Donor Infection. Cel Rep. 27 (13), 3780–3789. 10.1016/j.celrep.2019.05.103 PMC6693498 31242412

[B11] Chen X. Chen Y. Xin H. Wan T. Ping Y. (2020). Near-infrared Optogenetic Engineering of Photothermal nanoCRISPR for Programmable Genome Editing. Proc. Natl. Acad. Sci. USA 117 (5), 2395–2405. 10.1073/pnas.1912220117 31941712 PMC7007568

[B12] Chi J. Zhao J. Wei S. Li Y. Zhi J. Wang H. (2021). A CRISPR-Cas9-Based Near-Infrared Upconversion-Activated DNA Methylation Editing System. ACS Appl. Mater. Inter. 13 (5), 6043–6052. 10.1021/acsami.0c21223 33525876

[B13] Croce A. C. Bottiroli G. (2014). Autofluorescence Spectroscopy and Imaging: a Tool for Biomedical Research and Diagnosis. Eur. J. Histochem. 58 (4), 2461. 10.4081/ejh.2014.2461 25578980 PMC4289852

[B14] Deng W. Shi X. Tjian R. Lionnet T. Singer R. H. (2015). CASFISH: CRISPR/Cas9-mediated *In Situ* Labeling of Genomic Loci in Fixed Cells. Proc. Natl. Acad. Sci. USA 112 (38), 11870–11875. 10.1073/pnas.1515692112 26324940 PMC4586837

[B15] Deng S. Li X. Liu S. Chen J. Li M. Chew S. Y. (2020). Codelivery of CRISPR-Cas9 and chlorin e6 for spatially controlled tumor-specific gene editing with synergistic drug effects. Sci. Adv. 6 (29), b4005. 10.1126/sciadv.abb4005 PMC7439618 32832641

[B16] Devkota S. (2018). The Road Less Traveled: Strategies to Enhance the Frequency of Homology-Directed Repair (HDR) for Increased Efficiency of CRISPR/Cas-mediated Transgenesis. BMB Rep. 51 (9), 437–443. 10.5483/bmbrep.2018.51.9.187 30103848 PMC6177507

[B17] Doetschman T. Georgieva T. (2017). Gene Editing with CRISPR/Cas9 RNA-Directed Nuclease. Circ. Res. 120 (5), 876–894. 10.1161/CIRCRESAHA.116.309727 28254804

[B18] Feng X. Peng C. Chen Y. Liu X. Feng X. He Y. (2017). Discrimination of CRISPR/Cas9-induced Mutants of Rice Seeds Using Near-Infrared Hyperspectral Imaging. Sci. Rep. 7 (1), 15934. 10.1038/s41598-017-16254-z 29162881 PMC5698449

[B85] Fu Y. Rocha P. P. Luo V. M. Raviram R. Deng Y. Mazzoni E. O. (2016). CRISPR-dCas9 and sgRNA Scaffolds Enable Dual-Colour Live Imaging of Satellite Sequences and Repeat-Enriched Individual Loci. Nat. Commun. 7, 11707. 10.1038/ncomms11707 27222091 PMC4894952

[B19] Glass Z. Lee M. Li Y. Xu Q. (2018). Engineering the Delivery System for CRISPR-Based Genome Editing. Trends Biotechnol. 36 (2), 173–185. 10.1016/j.tibtech.2017.11.006 29305085 PMC5801045

[B20] Gootenberg J. S. Abudayyeh O. O. Lee J. W. Essletzbichler P. Dy A. J. Joung J. (2017). Nucleic Acid Detection with CRISPR-Cas13a/C2c2. Science 356 (6336), 438–442. 10.1126/science.aam9321 28408723 PMC5526198

[B21] Gu B. Posfai E. Rossant J. (2018). Efficient Generation of Targeted Large Insertions by Microinjection into Two-Cell-Stage Mouse Embryos. Nat. Biotechnol. 36 (7), 632–637. 10.1038/nbt.4166 29889212

[B22] Guan J. Liu H. Shi X. Feng S. Huang B. (2017). Tracking Multiple Genomic Elements Using Correlative CRISPR Imaging and Sequential DNA FISH. Biophys. J. 112 (6), 1077–1084. 10.1016/j.bpj.2017.01.032 28355536 PMC5375138

[B23] Guo H. Kommidi H. Vedvyas Y. McCloskey J. E. Zhang W. Chen N. (2019). A Fluorescent, [18F]-Positron-Emitting Agent for Imaging Prostate-specific Membrane Antigen Allows Genetic Reporting in Adoptively Transferred, Genetically Modified Cells. ACS Chem. Biol. 14 (7), 1449–1459. 10.1021/acschembio.9b00160 31120734 PMC6775626

[B24] He S. Song J. Qu J. Cheng Z. (2018). Crucial Breakthrough of Second Near-Infrared Biological Window Fluorophores: Design and Synthesis toward Multimodal Imaging and Theranostics. Chem. Soc. Rev. 47 (12), 4258–4278. 10.1039/c8cs00234g 29725670

[B25] He S. Chen S. Li D. Wu Y. Zhang X. Liu J. (2019). High Affinity to Skeleton Rare Earth Doped Nanoparticles for Near-Infrared II Imaging. Nano Lett. 19 (5), 2985–2992. 10.1021/acs.nanolett.9b00140 30983358

[B26] Hille F. Richter H. Wong S. P. Bratovič M. Ressel S. Charpentier E. (2018). The Biology of CRISPR-Cas: Backward and Forward. Cell 172 (6), 1239–1259. 10.1016/j.cell.2017.11.032 29522745

[B27] Hong G. Lee J. C. Jha A. Diao S. Nakayama K. H. Hou L. (2014). Near-infrared II Fluorescence for Imaging Hindlimb Vessel Regeneration with Dynamic Tissue Perfusion Measurement. Circ. Cardiovasc. Imaging 7 (3), 517–525. 10.1161/CIRCIMAGING.113.000305 24657826 PMC4079035

[B28] Hong Y. Lu G. Duan J. Liu W. Zhang Y. (2018). Comparison and Optimization of CRISPR/dCas9/gRNA Genome-Labeling Systems for Live Cell Imaging. Genome Biol. 19 (1), 39. 10.1186/s13059-018-1413-5 29566733 PMC5863892

[B29] Huang J. Pu K. (2020). Activatable Molecular Probes for Second Near‐Infrared Fluorescence, Chemiluminescence, and Photoacoustic Imaging. Angew. Chem. Int. Ed. 59 (29), 11717–11731. 10.1002/anie.202001783 32134156

[B30] Jiang F. Doudna J. A. (2017). CRISPR-Cas9 Structures and Mechanisms. Annu. Rev. Biophys. 46, 505–529. 10.1146/annurev-biophys-062215-010822 28375731

[B31] Kim K. Ryu S.-M. Kim S.-T. Baek G. Kim D. Lim K. (2017). Highly Efficient RNA-Guided Base Editing in Mouse Embryos. Nat. Biotechnol. 35 (5), 435–437. 10.1038/nbt.3816 28244995

[B32] Knott G. J. Doudna J. A. (2018). CRISPR-cas Guides the Future of Genetic Engineering. Science 361 (6405), 866–869. 10.1126/science.aat5011 30166482 PMC6455913

[B33] Kwon Y.-W. Ahn H.-S. Lee J.-W. Yang H.-M. Cho H.-J. Kim S. J. (2021). HLA DR Genome Editing with TALENs in Human iPSCs Produced Immune-Tolerant Dendritic Cells. Stem Cell Int. 2021, 8873383. 10.1155/2021/8873383 PMC8163544 34093711

[B34] Li J. Pu K. (2019). Development of Organic Semiconducting Materials for Deep-Tissue Optical Imaging, Phototherapy and Photoactivation. Chem. Soc. Rev. 48 (1), 38–71. 10.1039/c8cs00001h 30387803

[B35] Li L. Yang Z. Zhu S. He L. Fan W. Tang W. (2019). A Rationally Designed Semiconducting Polymer Brush for NIR‐II Imaging‐Guided Light‐Triggered Remote Control of CRISPR/Cas9 Genome Editing. Adv. Mater. 31 (21), 1901187. 10.1002/adma.201901187 30957918

[B36] Li C. Chen G. Zhang Y. Wu F. Wang Q. (2020a). Advanced Fluorescence Imaging Technology in the Near-Infrared-II Window for Biomedical Applications. J. Am. Chem. Soc. 142 (35), 14789–14804. 10.1021/jacs.0c07022 32786771

[B37] Li C. Li W. Liu H. Zhang Y. Chen G. Li Z. (2020b). An Activatable NIR‐II Nanoprobe for *In Vivo* Early Real‐Time Diagnosis of Traumatic Brain Injury. Angew. Chem. Int. Ed. 59 (1), 247–252. 10.1002/anie.201911803 31626380

[B38] Li X. Liang X. Yin J. Lin W. (2021). Organic Fluorescent Probes for Monitoring Autophagy in Living Cells. Chem. Soc. Rev. 50 (1), 102–119. 10.1039/d0cs00896f 33155002

[B39] Li H. Yang Y. Hong W. Huang M. Wu M. Zhao X. (2020). Applications of Genome Editing Technology in the Targeted Therapy of Human Diseases: Mechanisms, Advances and Prospects. Sig Transduct. Target. Ther. 5 (1), 1. 10.1038/s41392-019-0089-y PMC6946647 32296011

[B40] Li L. Hu S. Chen X. (2018). Non-viral Delivery Systems for CRISPR/Cas9-based Genome Editing: Challenges and Opportunities. Biomaterials 171, 207–218. 10.1016/j.biomaterials.2018.04.031 29704747 PMC5944364

[B41] Li Z. Zhao J. Muhammad N. Wang D. Mao Q. Xia H. (2018). Establishment of a HEK293 Cell Line by CRISPR/Cas9-mediated Luciferase Knock-In to Study Transcriptional Regulation of the Human SREBP1 Gene. Biotechnol. Lett. 40 (11-12), 1495–1506. 10.1007/s10529-018-2608-2 30232659

[B42] Liu J. Chang J. Jiang Y. Meng X. Sun T. Mao L. (2019). Fast and Efficient CRISPR/Cas9 Genome Editing *In Vivo* Enabled by Bioreducible Lipid and Messenger RNA Nanoparticles. Adv. Mater. 31 (33), 1902575. 10.1002/adma.201902575 PMC6732788 31215123

[B43] Liu W. Rudis M. R. Cheplick M. H. Millwood R. J. Yang J.-P. Ondzighi-Assoume C. A. (2020). Lipofection-mediated Genome Editing Using DNA-free Delivery of the Cas9/gRNA Ribonucleoprotein into Plant Cells. Plant Cel Rep. 39 (2), 245–257. 10.1007/s00299-019-02488-w 31728703

[B44] Liu Z. Chen S. Jia Y. Shan H. Chen M. Song Y. (2021). Efficient and High-Fidelity Base Editor with Expanded PAM Compatibility for Cytidine Dinucleotide. Sci. China Life Sci. 64, 1355–1367. 10.1007/s11427-020-1775-2 33420918

[B45] Lu S. Wang D. Hou Y. Guo D. Deng Y. Zhang X.-E. (2021). Illuminating Single Genomic Loci in Live Cells by Reducing Nuclear Background Fluorescence. Sci. China Life Sci. 64 (5), 667–677. 10.1007/s11427-020-1794-2 33131028

[B46] Lyu Y. He S. Li J. Jiang Y. Sun H. Miao Y. (2019). A Photolabile Semiconducting Polymer Nanotransducer for Near‐Infrared Regulation of CRISPR/Cas9 Gene Editing. Angew. Chem. Int. Ed. 58 (50), 18197–18201. 10.1002/anie.201909264 31566854

[B47] Ma H. Tu L.-C. Naseri A. Huisman M. Zhang S. Grunwald D. (2016). Multiplexed Labeling of Genomic Loci with dCas9 and Engineered sgRNAs Using CRISPRainbow. Nat. Biotechnol. 34 (5), 528–530. 10.1038/nbt.3526 27088723 PMC4864854

[B48] Ma H. Tu L.-C. Naseri A. Chung Y.-C. Grunwald D. Zhang S. (2018). CRISPR-sirius: RNA Scaffolds for Signal Amplification in Genome Imaging. Nat. Methods 15 (11), 928–931. 10.1038/s41592-018-0174-0 30377374 PMC6252086

[B49] Maass P. G. Barutcu A. R. Shechner D. M. Weiner C. L. Melé M. Rinn J. L. (2018). Spatiotemporal Allele Organization by Allele-specific CRISPR Live-Cell Imaging (SNP-CLING). Nat. Struct. Mol. Biol. 25 (2), 176–184. 10.1038/s41594-017-0015-3 29343869 PMC5805655

[B50] Mao S. Ying Y. Wu X. Krueger C. J. Chen A. K. (2019). CRISPR/dual-FRET Molecular beacon for Sensitive Live-Cell Imaging of Non-repetitive Genomic Loci. Nucleic Acids Res. 47 (20), e131. 10.1093/nar/gkz752 31504824 PMC6847002

[B51] Moreno A. M. Fu X. Zhu J. Katrekar D. Shih Y.-R. V. Marlett J. (2020). *In Situ* Gene Therapy via AAV-CRISPR-Cas9-Mediated Targeted Gene Regulation. Mol. Ther. 28 (8), 1931. 10.1016/j.ymthe.2020.06.027 32603653 PMC7403451

[B52] Muntean B. S. Zucca S. MacMullen C. M. Dao M. T. Johnston C. Iwamoto H. (2018). Interrogating the Spatiotemporal Landscape of Neuromodulatory GPCR Signaling by Real-Time Imaging of cAMP in Intact Neurons and Circuits. Cel Rep. 22 (1), 255–268. 10.1016/j.celrep.2017.12.022 PMC5761078 29298426

[B53] Nami F. Basiri M. Satarian L. Curtiss C. Baharvand H. Verfaillie C. (2018). Strategies for *In Vivo* Genome Editing in Nondividing Cells. Trends Biotechnol. 36 (8), 770–786. 10.1016/j.tibtech.2018.03.004 29685818

[B54] Neguembor M. V. Sebastian-Perez R. Aulicino F. Gomez-Garcia P. A. Cosma M. P. Lakadamyali M. (2018). (Po)STAC (Polycistronic SunTAg Modified CRISPR) Enables Live-Cell and Fixed-Cell Super-resolution Imaging of Multiple Genes. Nucleic Acids Res. 46 (5), e30. 10.1093/nar/gkx1271 29294098 PMC5861460

[B55] Nicholas J. Chen H. Liu K. Venu I. Bolser D. Saleh N. B. (2019). Utilization of Near Infrared Fluorescence Imaging to Track and Quantify the Pulmonary Retention of Single-Walled Carbon Nanotubes in Mice. NanoImpact 14, 100167. 10.1016/j.impact.2019.100167 32818159 PMC7430926

[B56] Ostrominski J. W. Yada R. C. Sato N. Klein M. Blinova K. Patel D. (2020). CRISPR/Cas9‐mediated Introduction of the Sodium/iodide Symporter Gene Enables Noninvasive *In Vivo* Tracking of Induced Pluripotent Stem Cell‐Derived Cardiomyocytes. Stem Cell Transl. Med. 9 (10), 1203–1217. 10.1002/sctm.20-0019 PMC7519772 32700830

[B57] Pan Y. Yang J. Luan X. Liu X. Li X. Yang J. (2019). Near-infrared Upconversion-Activated CRISPR-Cas9 System: A Remote-Controlled Gene Editing Platform. Sci. Adv. 5 (4), v7199. 10.1126/sciadv.aav7199 PMC6447385 30949579

[B58] Peng H. Le C. Wu J. Li X.-F. Zhang H. Le X. C. (2020). A Genome-Editing Nanomachine Constructed with a Clustered Regularly Interspaced Short Palindromic Repeats System and Activated by Near-Infrared Illumination. ACS Nano 14 (3), 2817–2826. 10.1021/acsnano.9b05276 32048826

[B59] Petolino J. F. (2015). Genome Editing in Plants via Designed Zinc finger Nucleases. *In Vitro* Cell.Dev.Biol.-Plant 51 (1), 1–8. 10.1007/s11627-015-9663-3 25774080 PMC4352198

[B60] Pickar-Oliver A. Gersbach C. A. (2019). The Next Generation of CRISPR-Cas Technologies and Applications. Nat. Rev. Mol. Cel Biol. 20 (8), 490–507. 10.1038/s41580-019-0131-5 PMC7079207 31147612

[B61] Qin P. Parlak M. Kuscu C. Bandaria J. Mir M. Szlachta K. (2017). Live Cell Imaging of Low- and Non-repetitive Chromosome Loci Using CRISPR-Cas9. Nat. Commun. 8, 14725. 10.1038/ncomms14725 28290446 PMC5424063

[B62] Qiu M. Glass Z. Xu Q. (2019). Nonviral Nanoparticles for CRISPR-Based Genome Editing: Is it Just a Simple Adaption of what Have Been Developed for Nucleic Acid Delivery? Biomacromolecules 20 (9), 3333–3339. 10.1021/acs.biomac.9b00783 31342740 PMC7261392

[B63] Rui Y. Wilson D. R. Green J. J. (2019). Non-Viral Delivery to Enable Genome Editing. Trends Biotechnol. 37 (3), 281–293. 10.1016/j.tibtech.2018.08.010 30278987 PMC6378131

[B64] Scully R. Panday A. Elango R. Willis N. A. (2019). DNA Double-Strand Break Repair-Pathway Choice in Somatic Mammalian Cells. Nat. Rev. Mol. Cel. Biol. 20 (11), 698–714. 10.1038/s41580-019-0152-0 PMC7315405 31263220

[B65] Seol J.-H. Shim E. Y. Lee S. E. (2018). Microhomology-mediated End Joining: Good, Bad and Ugly. Mutat. Res./Fundam. Mol. Mech. Mutagen. 809, 81–87. 10.1016/j.mrfmmm.2017.07.002 PMC6477918 28754468

[B86] Shao S. Zhang W. Hu H. Xue B. Qin J. Sun C. (2016). Long-Term Dual-Color Tracking of Genomic Loci by Modified sgRNAs of the CRISPR/Cas9 System. Nucleic Acids Res. 44 (9), e86. 10.1093/nar/gkw066 26850639 PMC4872083

[B66] Su Y. Yu B. Wang S. Cong H. Shen Y. (2021). NIR-II Bioimaging of Small Organic Molecule. Biomaterials 271, 120717. 10.1016/j.biomaterials.2021.120717 33610960

[B67] Sun N.-H. Chen D.-Y. Ye L.-P. Sheng G. Gong J.-J. Chen B.-H. (2020). CRISPR-Sunspot: Imaging of Endogenous Low-Abundance RNA at the Single-Molecule Level in Live Cells. Theranostics 10 (24), 10993–11012. 10.7150/thno.43094 33042266 PMC7532675

[B68] Tang H. Xu X. Chen Y. Xin H. Wan T. Li B. (2021). Reprogramming the Tumor Microenvironment through Second‐Near‐Infrared‐Window Photothermal Genome Editing of PD‐L1 Mediated by Supramolecular Gold Nanorods for Enhanced Cancer Immunotherapy. Adv. Mater. 33 (12), 2006003. 10.1002/adma.202006003 33538047

[B69] Tao Y. Yi K. Hu H. Shao D. Li M. (2021). Coassembly of Nucleus-Targeting Gold Nanoclusters with CRISPR/Cas9 for Simultaneous Bioimaging and Therapeutic Genome Editing. J. Mater. Chem. B 9 (1), 94–100. 10.1039/d0tb01925a 33220661

[B70] Tasan I. Sustackova G. Zhang L. Kim J. Sivaguru M. HamediRad M. (2018). CRISPR/Cas9-mediated Knock-In of an Optimized TetO Repeat for Live Cell Imaging of Endogenous Loci. Nucleic Acids Res. 46 (17), e100. 10.1093/nar/gky501 29912475 PMC6158506

[B71] Vu T. Doan D. Kim J. Sung Y. W. Tran M. Song Y. J. (2021). CRISPR/Cas‐based Precision Genome Editing via Microhomology‐mediated End Joining. Plant Biotechnol. J. 19 (2), 230–239. 10.1111/pbi.13490 33047464 PMC7868975

[B72] Wang S. Su J.-H. Zhang F. Zhuang X. (2016). An RNA-Aptamer-Based Two-Color CRISPR Labeling System. Sci. Rep. 6, 26857. 10.1038/srep26857 27229896 PMC4882555

[B73] Wang H.-X. Li M. Lee C. M. Chakraborty S. Kim H.-W. Bao G. (2017). CRISPR/Cas9-Based Genome Editing for Disease Modeling and Therapy: Challenges and Opportunities for Nonviral Delivery. Chem. Rev. 117 (15), 9874–9906. 10.1021/acs.chemrev.6b00799 28640612

[B74] Wang P. Zhang L. Zheng W. Cong L. Guo Z. Xie Y. (2018). Thermo-triggered Release of CRISPR-Cas9 System by Lipid-Encapsulated Gold Nanoparticles for Tumor Therapy. Angew. Chem. Int. Ed. 57 (6), 1491–1496. 10.1002/anie.201708689 29282854

[B75] Wang H. Nakamura M. Abbott T. R. Zhao D. Luo K. Yu C. (2019). CRISPR-mediated Live Imaging of Genome Editing and Transcription. Science 365 (6459), 1301–1305. 10.1126/science.aax7852 31488703 PMC13030913

[B76] Wang M. Chen K. Wu Q. Peng R. Zhang R. Li J. (2020). RCasFISH: CRISPR/dCas9-Mediated *In Situ* Imaging of mRNA Transcripts in Fixed Cells and Tissues. Anal. Chem. 92 (3), 2468–2475. 10.1021/acs.analchem.9b03797 31782306

[B77] Wu X. Mao S. Yang Y. Rushdi M. N. Krueger C. J. Chen A. K. (2018). A CRISPR/molecular beacon Hybrid System for Live-Cell Genomic Imaging. Nucleic Acids Res. 46 (13), e80. 10.1093/nar/gky304 29718399 PMC6061827

[B88] Xu C. F. Chen G. J. Luo Y. L. Zhang Y. Zhao G. Lu Z. D. (2021). Rational Designs of *in vivo* CRISPR-Cas Delivery Systems. Adv. Drug Deliv. Rev. 168, 3–29. 10.1016/j.addr.2019.11.005 31759123

[B78] Yang L.-Z. Wang Y. Li S.-Q. Yao R.-W. Luan P.-F. Wu H. (2019). Dynamic Imaging of RNA in Living Cells by CRISPR-Cas13 Systems. Mol. Cel. 76 (6), 981–997. 10.1016/j.molcel.2019.10.024 31757757

[B79] Yang Y. Yu Y. Chen H. Meng X. Ma W. Yu M. (2020). Illuminating Platinum Transportation while Maximizing Therapeutic Efficacy by Gold Nanoclusters via Simultaneous Near-Infrared-I/II Imaging and Glutathione Scavenging. ACS Nano 14 (10), 13536–13547. 10.1021/acsnano.0c05541 32924505

[B80] Zetsche B. Gootenberg J. S. Abudayyeh O. O. Slaymaker I. M. Makarova K. S. Essletzbichler P. (2015). Cpf1 Is a Single RNA-Guided Endonuclease of a Class 2 CRISPR-Cas System. Cell 163 (3), 759–771. 10.1016/j.cell.2015.09.038 26422227 PMC4638220

[B81] Zhang J.-P. Li X.-L. Li G.-H. Chen W. Arakaki C. Botimer G. D. (2017). Efficient Precise Knockin with a Double Cut HDR Donor after CRISPR/Cas9-Mediated Double-Stranded DNA Cleavage. Genome Biol. 18 (1), 35. 10.1186/s13059-017-1164-8 28219395 PMC5319046

[B82] Zhang X. Wang W. Su L. Ge X. Ye J. Zhao C. (2021). Plasmonic-Fluorescent Janus Ag/Ag2S Nanoparticles for *In Situ* H2O2-Activated NIR-II Fluorescence Imaging. Nano Lett. 21 (6), 2625–2633. 10.1021/acs.nanolett.1c00197 33683889

[B83] Zhao M. Li B. Fan Y. Zhang F. (2019). *In Vivo* Assembly and Disassembly of Probes to Improve Near‐Infrared Optical Bioimaging. Adv. Healthc. Mater. 8 (13), 1801650. 10.1002/adhm.201801650 31094099

[B84] Zinovkina L. A. (2018). Mechanisms of Mitochondrial DNA Repair in Mammals. Biochem. Mosc. 83 (3), 233–249. 10.1134/S0006297918030045 29625543

